# Interferometric scattering for optical tomoslicing of transparent solids

**DOI:** 10.1038/s41377-026-02344-z

**Published:** 2026-06-30

**Authors:** Yuan Chai, Hong-Hua Fang, Zhen-Ze Li, Tian-Wei Wang, Shao-Feng Liu, Hong-Ren Chen, Shu-Chang Li, Xiao-Yan Li, Jia-Ming Lyu, Hong-Bo Sun

**Affiliations:** 1 State Key Laboratory of Precision Measurement Technology and Instruments, Department of Precision Instrument, Tsinghua University, Beijing, 100084 China; 2 Key Laboratory of Advanced Light Conversion Materials and Biophotonics, School of Chemistry and Life Resources, Renmin University of China, Beijing, 100872 China; 3 Mechano-X Institute, Applied Mechanics Laboratory, Department of Engineering Mechanics, Tsinghua University, Beijing, 100084 China

**Keywords:** Laser material processing, Nonlinear optics, Nanophotonics and plasmonics

## Abstract

While light scattering is widely utilized in optical metrology and measurement, it has long been regarded as detrimental in laser-material processing. Here, we report an interferometric scattering effect that overturns this conventional view by resolving the six-decade challenge of axial resolution in optical manufacturing. This breakthrough elevates the axial resolution from micrometers, e.g., ~2 µm in transparent solids slicing, to the sub-10 nm level. The underlying mechanism involves the controlled sequential generation of nano-scatterers through interference between the incident laser and deliberately seeded scattering centers. Based on this phenomenon, we developed an interferometric scattering-based optical tomoslicing technology (*i*-SOT), achieving kerf widths as narrow as 7 nm under an industrial standard efficiency of up to 400 mm²/s. This unprecedented axial resolution enables nearly lossless laser wafering from ingots—reducing mass loss from ~30% to below 1% — with transformative potential for manufacturing laser crystals, photovoltaics, and microelectronic chips.

## Introduction

Light scattering occurs when light interacts with particles or local inhomogeneities in a medium, causing its path to deviate from a straight trajectory. This phenomenon underlies everyday observations such as the blue sky, white clouds, and crimson sunsets, and it has enabled important applications in optical measurement and metrology—including material identification^1,2^, biomedical imaging^3,4^, environmental monitoring^5^, and lidar systems^6,7^. In these applications, the scattered light field, E_sca_(r), carries local information via intensity, phase, polarization, or spectral composition. Yet, in high-resolution microscopy and precision laser manufacturing, scattering is often a nuisance. It not only restricts penetration depth but also severely degrades resolution.

Enhancing resolution, whether in optical imaging or manufacturing, represents a fundamental scientific and technological pursuit. In optical imaging, techniques such as structured illumination microscopy^8^ and stimulated emission depletion (STED) microscopy^9^ have pushed lateral resolutions to the scale of tens of nanometers. In laser processing, although lasers were applied to material processing soon after their invention, it was the advent of femtosecond laser writing that substantially improved efficiency and feature fineness, enabling sub-diffraction-limit spatial resolutions as fine as 120 nm via nonlinear effects^10^. Nevertheless, achieving true nanoscale precision, particularly in cutting and slicing of hard materials, remains a formidable challenge for state-of-the-art laser technologies. Recent advances in laser-based processing, such as optical near-field techniques (e.g., optical far-field-induced near-field breakdown, O-FIB^11^), can produce surface features as small as 10 nm. However, these approaches are generally confined to surface or near-surface regions and emphasize lateral resolution. In contrast, axial resolution, which is critical for applications such as semiconductor wafer dicing, has received far less attention, despite being equally, if not more, important. As a result, axial resolution remains a persistent limitation in deep, high‑precision machining.

This limitation in axial resolution arises from fundamental optical constraints. The limited range of incident angles (θ < 180°) and the inherent nonlinear effects in lens-based focusing systems inevitably lead to a non-spherical point spread function, resulting in a focal spot that is much elongated along the axial direction compared to the lateral direction. Currently, the best-reported axial resolution in laser-based processing is approximately 150 nm^12,13^, achievable only with high-numerical-aperture (0.95, or higher-NA) objectives, which have limited working distances and are restricted to near-surface regions. This constraint is particularly evident in applications such as laser wafering, where deep penetration into crystalline materials is required, rendering high-NA optics impractical. Under these conditions, the combined effects of scattering and other factors typically yield kerf widths of several micrometers. Specifically, for slicing crystalline materials, the current state-of-the-art axial resolution is about 2 µm under laboratory conditions^14,15^, whereas industrial processes generally achieve only around 50 µm^16^.

In this work, we introduce interferometric-scattering-enabled optical tomoslicing (i-SOT), a technique that enables laser-based whole-plane cutting with nanometer precision perpendicular to the light’s propagation. Departing from the conventional view that treats scattering as a detrimental effect in laser processing, we harness scattered light to achieve localized energy deposition for permanent material modification and unprecedented axial resolution. In *i*-SOT, irreversible electron excitation, the core process of optical manufacturing, occurs only when the incident light intensity is sufficiently high, as described by the relation |***E***_sca_ | ^2^ = *α* |***E***_ref_ | ^2^. A key aspect of this principle lies in the controlled use of scatterers and their directional propagation via cascaded secondary scattering events. This approach reduces the interferometric scattering site and feature sizes by more than two orders of magnitude, overcoming traditional limits set by the incident angle (θ < 180°) and the non-spherical point-spread function of focusing lenses (SI section 1). Experimentally, we show that i-SOT can achieve a kerf width as narrow as 7 nm, significantly exceeding current capabilities. It is expected that i-SOT will pave the way for revolutionary industrial laser-machining technologies, especially for applications requiring kerf-free, large-scale wafering.

## Results

### Interferometric scattering effect

Different from imaging and sensing, where interferometric scattering is initiated from randomly distributed nano-scatterers including nanoparticles, macromolecules, or local inhomogeneities in the focal volume, *i*-SOT resorts to the intentionally induced nanopore as an initial scatterer. It has been well established that nanopores of all dimensions less than a hundred of nanometers are created due to micro-explosion by focused femtosecond laser excitation of various transparent solids, from glassy to crystalline phases^17–21^. Because the excitation of materials at the focal point center is so strong, a high-density electron-ion plasma occurs, which then releases the deposited energy in an explosive way. This leads to complete cleavage of covalent bonds in the core volume, and thus forms core-shell-like nanopores^22,23^. Provided nanopores be closely arrayed, they readily produce nanometric lateral slicing plane. However, this goal is hindered by the neighboring effect, that is, new nanopores are not possibly generated near a pre-existingone since the latter tends to scatter off the incident energy so that the remaining energy becomes insufficient to produce any material damage. A reasonably large separation, e.g., several times of the initial pore size, is considered as a prerequisite for a neighboring nanopore formation. A modificative layer containing such sparsely distributed nanopores is not mechanically weak enough for femtosecond laser machining.

**Fig. 1:**
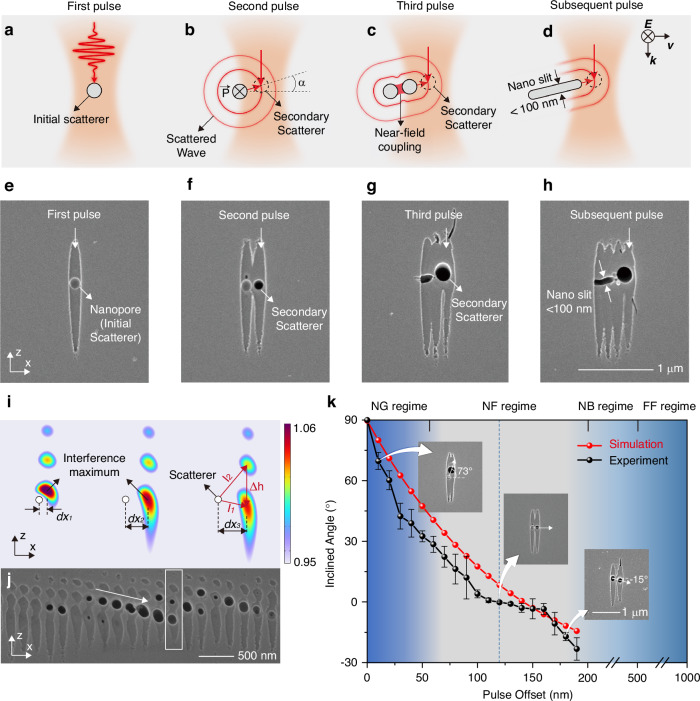
Interferometric scattering effect. **a**–**d** Schematic production of initial and secondary scatterers. The initial scatterer, a nanopore, is purposely induced by femtosecond laser induced micro-explosion, from which the scattered light interferes with the incident light, producing a secondary scatterer. The 3^rd^ scatter is then created by the interferometric scattering between the 2^nd^ scatter and the incidence. The wave vector *k* is oriented vertically downward, the polarization direction **E** is perpendicular to the paper plane, and the laser spot moves to the right. **e**–**h** Scanning electron microscopic images. Evolution of the structure inside silica at a depth of 50 μm. The lateral offset is δ = 100 nm, with the wave vector, polarization, and spot movement directions consistent with the schematic. **i** Simulation of interferometric scattering effect from a nanopore. The initial and secondary scatterers with varied offsets of 40 nm, 140 nm, and 180 nm. The intensity in the figure is relative intensity, with the incident field intensity being 1 and the unit of color bar is 1. **j** An example of the nanopore propagation with a lateral offset 180 nm. **k** The relationship between tilt angle, *α*, of the secondary nanopore relative to its seed and the lateral offset

An unexpected phenomenon was observed when the incident laser approached a pre-formed nanopore as the initial scatterer within a critical distance, e.g., < 200 nm (Fig. 1a). The scattered light interferes coherently with the undisturbed portion of the incident beam (Fig. 1b), producing a localized intensity maximum that induces a secondary nanopore (Fig. 1b). By slightly shifting the incident laser laterally relative to an existing scatterer, secondary scatterers are consecutively generated (Fig. 1c, d). Experimentally, a 515 nm pulsed laser with a pulse width of 230 fs was focused into fused silica as the testing material through an objective lens with NA = 0.6. An 80 nm diameter spherical nanopore is produced as expected as the initial scatterers (Fig. 1e), and a 100 nm lateral offset, *δ*, leads to a secondary nanopore of similar shape and size (Fig. 1f). This secondary nanopore subsequently functions as a new scattering center, enabling the formation of a third nanopore (Fig. 1g), and the process proceed iteratively (Fig. 1h).

The above process is readily recognized as the interferometric scattering effect but with the nanopores instead of nanoparticles as the scatterers. The validity of the nanopores as scattering centers is then examined by a numerical electromagnetic calculation using COMSOL Multiphysics. In the core-shell-like model, the nanopores were assigned a slightly reduced refractive index relative to the surrounding matrix^23^, as is reasonable since the core volume consists of diluted material due to micro-explosion. When irradiated by a laser beam that is laterally offset from the scattering center, each nanopore functions as an excited electric dipole, oscillating along the polarization direction (perpendicular to the plane of the paper). The near-field component dominates the dipole radiation^24^ since the nanopore size is much smaller than the wavelength (< *λ*/10), which is then expressed as:

$${{\boldsymbol{E}}}_{sca}^{near}({\boldsymbol{r}},t)=\frac{1}{4\pi \varepsilon }\frac{3\hat{{\boldsymbol{r}}}(\hat{{\boldsymbol{r}}}\cdot {\boldsymbol{p}})-{\boldsymbol{p}}}{{r}^{3}}{e}^{i(kr-\omega t)}$$

where ***p*** = *γ****E***_inc_ is the amplitude of the electric dipole vector, and *γ* is the polarizability.

The simulation confirms that scattered light does interfere constructively with the incident beam, producing a series of intensity maxima along the optical axis (Fig. 1i). The primary (0^th^-order) speckle exhibits a sub-diffraction limit size and an intensity up to 10% higher than the incident beam, creating favorable conditions for secondary scatterer formation via micro-explosion. The lateral offset δ determines the position of the secondary scatterers within a critical range of δ < 200 nm. Experimentally, δ = 40 nm (Fig. 1i, left), δ = 140 nm (Fig. 1i, center), and δ = 180 nm (Fig. 1i, right) all produce secondary scatterers of intervals equal to the offsets.

It is interesting to notice from the simulation that high-order interference speckles always exist, and the relative distances among the initial scatterer, *0*^th^ and high-order speckles follows a phase condition: ∆*h* + *l*_2_ - *l*_1_ = *m*λ, where *m* denotes interference order, λ denotes the wavelength in the medium, ∆*h* denotes the longitudinal height difference between the extremum points of the interference field, and *l*_1_, *l*_2_ denote the distances from the nanoseed to the extreme points respectively. Assuming three-photon absorption process, δ = 40 nm and δ = 180 nm lead to 1^st^ order interference speckle intensity of 90.4% and 95.7% of the 0^th^ order (Fig. 1i). If the incident laser power is purposely increased, and the offset is chosen to 180 nm, near the upper limit of the critical range, high-order interference intensity maxima, up to *m* = 3 are all printed to material (Fig. 1j). These results unequivocally demonstrate the role of interferometric scattering in the sub-diffraction-limit formation of secondary nanopores.

The laser beam offset, δ, plays a critical role in the secondary nanopore induction and evolution, according to which four different light-matter interaction regimes are divided (Fig. 1k). (i) Far-field (FF) regime (δ > 500 nm), defined by the lateral diffraction limit, where micro-explosion occurs insusceptible to the existence of early produced scatterers; (ii) Neighbor (NB) regime (500 nm > δ > 200 nm), where neighboring effect dominates with δ between the far-field and near-field limits. Neither secondary nor isolated nanopores can be formed due to insufficient energy deposition; (iii) Near-field (NF) regime (δ < 200 nm). Scattering interference effect dominates and secondary nanopores are deterministically generated. (iv) Nanograting (NG) regime (δ < 50 nm) as a special case of regime III. In this case, secondary nanopores are subjected to disturbance and modulation by its scatterer, resulting in a spatially overlapped volume consisteing of nanogratings. The δ values categorizing different regimes are dependent on materials, but the existence of the four regimes is universal to various transparent solids.

The near-field regime with 50 nm < δ < 200 nm works for continuous production of secondary scatterers. Once δ is chosen, the vertical coordinate, or the orientation of a secondary nanopore, defined by the inclined angle, α, is spontaneously fixed (Fig. 1b). Technically it means the convenience determining a secondary nanopore orientation simply by the offset selection. Both simulation and experiments exhibit that scanning δ from 0 to 190 nm yields a monotonic decrease in α, from 90° to -25°. There are three special cases worthy of further discussion. The first case, δ = 0 nm and α = 90° corresponds to a secondary nanopore directly above the initial seed. This configuration enables backscattering interference as previously reported^25^, whereby vertical nanopore arrays facilitate high-aspect-ratio dicing (>10,000:1). The second case occurs when 0 > α > -20°, achieved by 120 nm < δ < 190 nm. The occurrence of forward interferometric scattering is completely out of our expectations when the backscattering interference model was built^25^. This, together with the fact of tilt slicing along any angle of (δ = 50 ~ 120 nm; α = 0 ~ 30°), means the unique capability of optical nanofabrication of complex 3D geometries in transparent solids by subtractive and modificative means. In contrast, such high accuracy fabrication was achieved only by an additive approach, for example, two-photon-induced photopolymerization^10,26–29^. The third case is α = 0° achieved under δ = 120 nm. Sequential lateral extension of secondary nanopores is exactly the conditions required for lateral slicing, laying the foundation for *i*-SOT-based wafering applications.

### Femtosecond laser writing by interferometric scattering effect

Femtosecond laser direct writing has been established as a powerful tool to manufacture complex 3D structures and devices for a wide variety of applications during the last decade. Despite sub-micrometer or even nanometer spatial resolution has been claimed, most of them denote lateral spatial resolution. Since any focal spot from a focusing lens takes intrinsically ellipsoid form with long axis parallel to the light beam direction, the vertical spatial resolution, although of the same importance for 3D fabrication as the lateral one, is much worse and hence less discussed. The interferometric scattering produces secondary nanopores by light maxima of more sphere shape from two-beam interference, which dramatically improves the vertical spatial resolution.

**Fig. 2:**
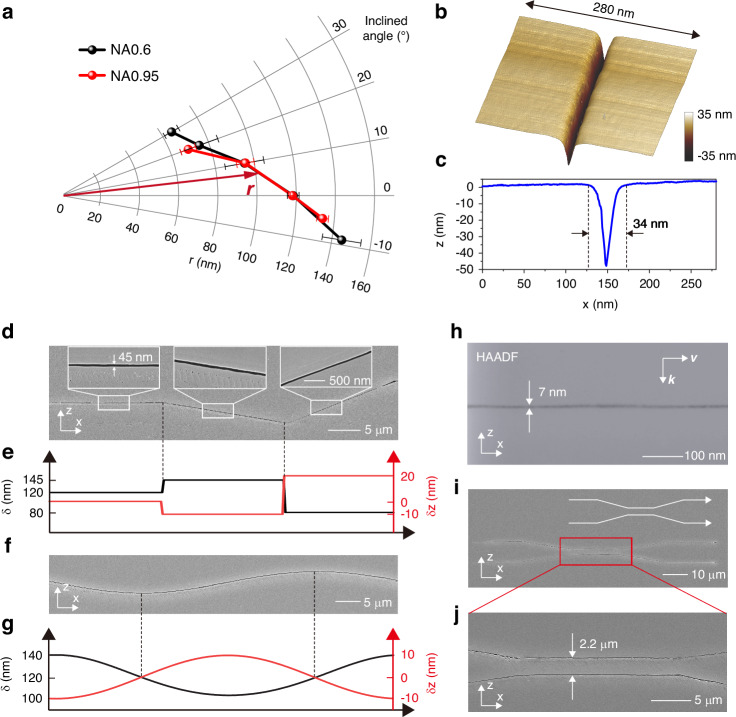
Femtosecond laser direct writing by interferometric scattering effect. **a** Polar plot showing the relationship between the nano-slit orientation, α, versus the laser beam offsets. **b**, **c** Atomic Force Microscopy (AFM) images of the nanoslit after slight etching. **d** Three-segment zigzag nano-slit structure with 3 magnified views. **e** Corresponding strategy of offsets design. **f** Curved nano-slit structure and **g** the corresponding offset design. **h** High-angle annular dark-field (HAADF) STEM image of a horizontal nano-slit. **i**, **j** Demonstration of nearest achievable nano-slit spacing, showing the hindrance of neighboring effect

Interferometric scattering effect allows for femtosecond laser scattering writing, i.e., complex 3D shapes are created by secondary nanopores propagation instead by focal spot depicting. In this circumstance, the laser beam offset, δ, simultaneously determines *x* and *z* coordinates of secondary nanopores, with the initial nanopore serving as the coordinate origin (Fig. 1k). This enables writing of arbitrary 3D trajectories within transparent solids through precise manipulation of δ, as exemplified by the work line illustrated in polar coordinates (Fig. 2a). This holds true only within localized regions where variations in δ do not significantly alter the light intensity distribution around existing scatterers.

For large-scale patterning, it is essential to dynamically adjust the laser focal spot along the desired trajectory to maintain a consistent scattering interference pattern. In this process, the lateral step size δ governs the orientation, *α* and z-coordinate of subsequent nanopores, while the vertical step size δ_z_ ensures consistent energy delivery for stable formation of secondary nanostructures.

The determination of δ_z_ requires an iterative optimization process since elevating the focal spot alters the scattering interference conditions. It is, however, a fast convergence process both experimentally and numerically, and isn’t challenging. A parameter set of δ = 120 nm and δ_z_ = 0 nm, denoted by (120, 0) produces a lateral straight line (Fig. 2d, left, e), while (143, -25) and (75, 27) lead to tilted straight lines of incident angles of -10° (Fig. 2d, middle, e) and 20° (Fig. 2d, right, e), respectively. It is not surprising that smooth, wavy lines (Fig. 2f) are produced by laser focal spot scanning with pinpoint (δ, δ_z_) setting (Fig. 2g). There are two points that are worth mentioning. First, a purposely chosen laser pulse energy destroys the wall between the secondary nanopore and its initial scatterer, and an internally connected inverse nanowire forms naturally following the scanning locus. 3D networks consisting of the inverse nanowires may find immediate applications in lab-on-a-chip systems, nanofluidic cooling structures for electronic and photonic chips, and biological filtering membranes. Second, the nanoscale feature is maintained after the secondary nanopore connection, as is proved by 34 nm wire width even after sample polishing and etching for atomic force microscopy (Fig. 2b, c). The smallest linewidth achieved experimentally is solely 7 nm (Fig. 2h) for the raw modified layer, which is magnified to 45 nm by wet etching for ease of SEM imaging. The minimum kerf width achieved experimentally is 7 nm in *i-*SOT wafering, as detailed later.

### Interferometric scattering-based optical tomoslicing (i-SOT)

**Fig. 3:**
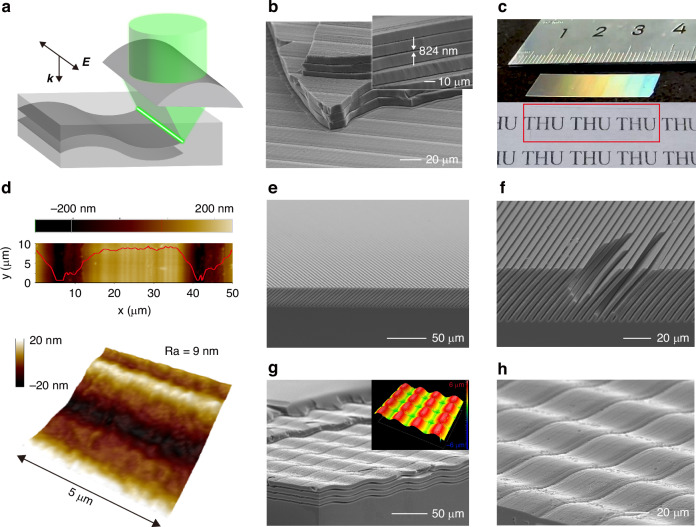
Interferometric scattering-based optical tomoslicing. **a** Schematic of line light field production by a cylindrical lens. **b** Planar slicing results showing a kerf width of 824 nm, local and magnified views. **c** Planar wafering with high transmittance using cold split without etching. The wafering size is 30×10 mm. **d** Surface flatness of the sliced surface. The overall roughness, Ra ~ 9 nm inside a line light field, and Ra ~160 nm considering the inter-field connection. **e**, **f** Tilted slicing with an inclination angle of ~45°. **g**, **h** Demonstration of wavy slicing. The peak-to-valley height difference is 12 μm, the period is 70 μm, and the minimum radius of curvature is approximately 21 μm

In simulations and experiments, we found that the aforementioned interference scattering model and nanoslit fabrication are applicable not only to a single spherical seed but also to a linear seed. We use a cylindrical lens (Fig. 3a) to produce a nanoslit, closely arrayed and inter-connected nanopores, as the initial scatterer. Surprisingly the initial nano-slit scatterer yields secondary nano-slits the same way as nanopores do. This enables a laser wire sawing tool to precisely slice a transparent solid into pieces. The ability is termed optical tomoslicing, which overcomes the limit of vertical spatial resolution by interferometric scattering, similar to the case of laser scattering writing discussed earlier. The entire technology is thus defined as *i*-SOT, i.e., interferometric scattering-based optical tomoslicing.

The *i*-SOT technology defines the slicing trace by pinpoint offset setting (Fig. 2a), too, instead by direct focal line scanning. Experimentally, the 515-nm-wavelength, 230-fs-pulsewidth laser beam was focused by a cylindrical lens with a focal length of 400 mm to generate a line optical field. The beam is then compressed and projected through a 4 *f* beam reduction system to create a line light field at its focal plane (Fig. 3a). The first unprecedented effect of *i*-SOT usage is the sub-micrometer kerf width (Fig. 3b). For example, the layer space in a horizontally sliced 10-μm-thick wafer is 824 nm, which is larger than the raw kerf value since it was widened by etching for the visibility under SEM. The i-SOT technology allows direct split^16^ after laser process with a kerf width of only 7 nm. As seen in Fig. 3c, a 30×10 mm slice was obtained (see methods). The small kerf naturally leads to smaller surface roughness, for example, Ra = 9 nm within a line field range, and 160 nm considering the inter-field seams (Fig. 3d). Both the kerf and surface roughness are more than two orders of magnitude superior to those by conventional wafering technologies, ~20 μm by laser-assisted approach, and ~40 μm in diamond- or slurry-based wire sawing. A wafer of 100-μm thickness with a kerf of submicron width denotes mass loss less than 1%, a level reasonably called kerf-free for crystal wafering.

Another unique advantage of *i*-SOT is the high processing efficiency inherent in the line-form, instead of point-form light field. With a cylinder lens of NA = 0.6, the light field can be transformed to to a width of 50 μm width and scanned at1 m/s velocity, as may be lifted by further extending the line field length to, for example, 20 mm, which accounts for a scanning efficiency of 0.02 m^2^/s. In addition, both the thin wafer thickness and small kerf width allow for consecutive multi-layer wafering. Different tilt angle wafering has been demonstrated. As an example, Fig. 3e, f show 45° angle tilt wafering. The largest possible layer number is restrained by the lens working distance and wafer thickness. Generally, 10-μm wafer thickness is safe for neighboring plane exposure, and under extreme condition, a 2.2-μm thickness has been achieved (Fig. 2i, j). As an extension of the case in Fig. 2f, where a wavy inverse nano-slit is demonstrated, curved-surface wafers of wavy cross-sections are attained by scanning with proper (δ, δ_z_) setting (Fig. 3g, h).

### Wafering of crystalline materials by *i-*SOT

The *i-*SOT technology has been approved valid using fused silica as the test material. There is no doubt that it is applicable to other transparent solids since interferometric scattering effect is universal upon femtosecond laser excitation. However, the inherent mechanical anisotropy induces competition between two mechanisms: secondary nanopore-governed slicing and local expansion-dominated cleavage along high-index crystalline planes. The 3D regular arraying of atoms categorizes crystals into 14 symmetry point groups and 230 space groups, leading to a relatively weak connection between low-index planes, as laying the foundation for semiconductor cleavage physics. In *i-*SOT of crystalline materials, laser-induced micro-explosion causes local expansion around nanopores or nano-slits, which serves not only as the scattering center for the secondary nanopores and nano-slits generation, but also the seeds of cleavage along low-index planes upon shockwave actions from subsequent laser pulses. It is obvious that slicing crystals by *i-*SOT along low-index plane tangent direction as done in common laser-assisted wafering, jointly aided by the two forces, would much facilitate the wafering process.

**Fig. 4:**
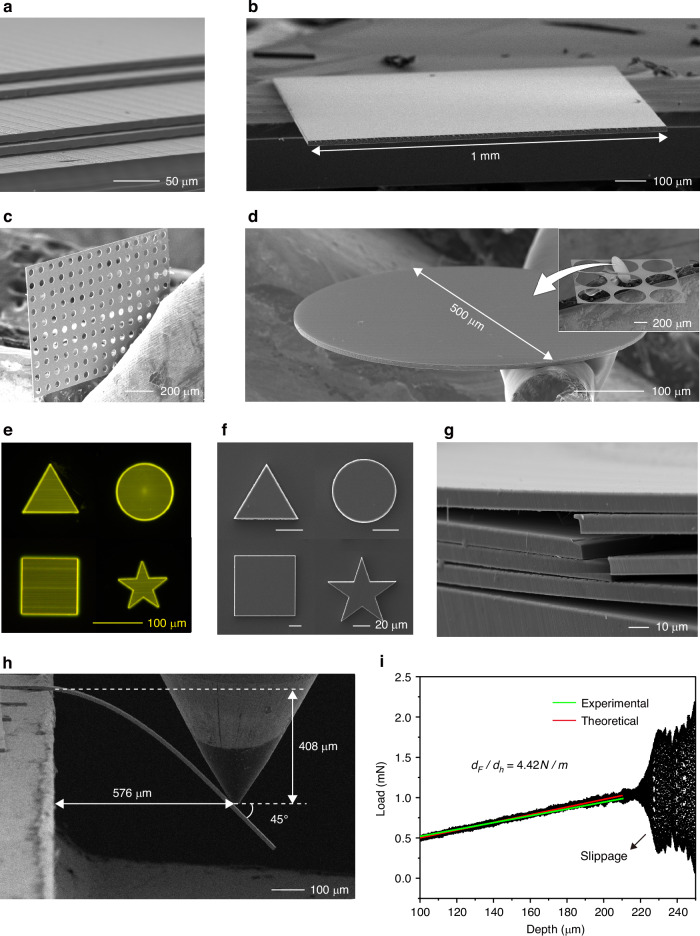
Wafering and fine machining of crystalline materials by *i*-SOT. **a** 4-layer slicing of pure YAG crystals. The wafer thickness is 8.6 μm and the kerf width is 0.9 μm. **b** Square YAG thin plate with a side length of 1 mm. **c** Nd: YAG thin-plate with a 10×15 holes array. The diameter of the holes is 30 µm. **d** Disk-shaped YAG thin plate with a diameter of 500 μm. **e** Fluorescence images of Ce: YAG slices with sdifferent shapes. **f** SEM micrographs of MgAl₂O₄ crystal slices with complex shapes. **g** Cross-sectional view of MgAl₂O₄ slices with a thickness of 9.5 μm. **h** Mechanical properties test of sliced YAG thin plate. Cantilever thickness: 9.55 μm, width: 30 μm, length: 1 mm. **i** Corresponding stress-strain curve demonstrating the robust mechanical integrity of these ultrathin YAG plates

Experimentally, YAG (Fig. 4a-e, h) and MgAl₂O₄ (Fig. 4f, g) crystals are processed by *i-*SOT along the (100) plane. In a sequential 4-layer YAG slicing of 8.6-μm wafer thickness and 0.9-μm kerf width, complete cleavage between layers is firmly approved by internal X-Ray imaging (Fig. S17d), as well as by the steplike planes of fracture in Fig.4a. The ease of spalling of *i-*SOT-sliced crystals is obviously observed from the random separation of the 4-layer wafers (Fig. 4g). Separated wafers allows for further processing by femtosecond laser, such as being cut into 1mm-side length square (Fig. 4b) or 0.5mm-diameter disk (Fig. 4d). 150 holes of regular 30-μm diameter are further drilled out of the YAG slab (Fig. 4c). Smaller structures of complicated geometries are also machined from the wafers of different crystals, Ce:YAG (Fig. 4e) and MgAl₂O₄ (Fig. 4f).

*i-*SOT wafering and subsequent laser fine machining of crystals cause negligible sub-surface and internal mechnical damage. To confirm this, a 1-mm long, 9.95-µm thick YAG slab cantilever was fabricated and subjected to a bending test with a diamond indenter. The cantilever exhibits excellent flexibility, as it reaches a maximum bending angle of 45° and a minimum radius of curvature of 271 µm before frictional slipping occurred at 220 µm displacement, causing oscillations in the force curve (Fig. 4i). The measured force-displacement slope was 4.42 N/m, closely matching the theoretical value of 4.24 N/m calculated from YAG’s Young’s modulus. No mechanical fatigue or performance degradation was detected after aging tests.

## Discussion

Efforts towards improving the vertical resolution in optical manufacturing are being hindered by the fact that focusing by any lenses results inevitably in a non-spherical point spread function, i.e., a longitudinal focal spot size much larger than that in the lateral direction. The interferometric scattering based optical tomoslicing achieves unprecedented longitudinal resolution down to 7 nm. This breakthrough significantly reduces kerf loss, lowering mass loss from approximately 30% to less than 1%, thereby reducing production costs for premium-quality crystalline thin slices. Moreover, this method is largely insensitive to NA of the focusing lenses; even at relatively low values, NA ~ 0.6, nanometric resolution is maintained while sufficient working distance for simultaneous processing of multiple layers is retained. We have consistently produced ultrathin wafers below 10 μm thickness across a variety of transparent materials, from glasses to crystalline solids. The broader applicability of this method to other materials requires more systematic study. As a unique lateral slicing technology, *i-*SOT holds great promise to advance material processing and applications spanning photovoltaics, laser crystals, and semiconductor chips.

## Materials and methods

### Laser processing system

The experiment utilized a Pharos laser for processing, with a pulse width of 230 fs, a wavelength of 515 nm, and a repetition rate of 10 kHz. The beam energy was controlled by an electronically adjustable attenuator (Altechna Watt Pilot), composed of a λ/2 zero-order waveplate and a thin-film polarizer. The beam spot, after expansion, had a diameter of approximately 10 mm. It was then focused into a line field using a cylindrical lens with f = 400 mm, followed by a beam reduction system consisting of an f = 500 mm lens and an Olympus 40×/0.6 focusing objective to compress and focus the line field, resulting in a line width of about 50 μm. The samples were translated with a three-axis high-resolution computer-controlled stage (Aerotech, ABL1500-XY, ANT130LZS).

The laser we used has relatively low power and can only achieve a 50 μm spot size at 200 kHz, resulting in an actual slicing efficiency of 1.2 mm²/s. Given that in industrial applications, for a femtosecond laser with a total power of 200 W (e.g., IceFyre FS IR200 laser), a single pulse energy of 200 μJ can be achieved at 1 MHz repetition rate. If this high-power laser were used, the theoretical slicing efficiency could reach 400 mm²/s.

### Sample preparation and processing

The fused silica used was JGS1 silica provided by Changchun-Jinlong Company. Pure YAG single crystal <100> and MgAl_2_O_4_ single crystal <100 > , with dimensions of 5×5×0.5 mm, were obtained from Hefei-Kejing Material Technology. The 0.2% doped Ce: YAG crystal had dimensions of 5×5×0.5 mm.

All samples were processed using the same method. After cleaning, the samples were fixed on a porous stone and leveled. The polarization direction was aligned with the linear spot direction. The displacement stage moved perpendicular to the polarization direction. The pulse interval was controlled by the movement speed of the displacement stage. For silica slicing, the required single-pulse energy was approximately 3.35 μJ. To ensure minimal overlap regions, the laser energy was adjusted based on depth and path spacing.

### Sample characterization and testing

To observe the cross-sectional morphology after laser scanning, the samples were sectioned and polished. For cutting, samples were cleaved after laser scribing, or processed using a Diamond Wire Saw (STX-402, SY-Kejing) for harder, non-cleavable materials. The exposed cross-sections were then mechanically polished using a Precision Polishing Machine (UNIPOL-802, SY-Kejing). To enhance the visibility of the cross-sectional features, selective etching was performed, since the laser modified region has higher etch selectivity. For silica, a 2% hydrofluoric acid (HF) solution was used; for YAG crystals, 30% phosphoric acid was applied, while MgAl₂O₄ crystals were etched with a mixture of hydrofluoric acid (HF) and nitric acid (HNO_3_). For direct split of wafering, cold split technology was used to apply external stress.

The sample morphology was characterized using a scanning electron microscope (SEM, IT-700HR, JEOL), while surface roughness was evaluated via atomic force microscopy (AFM, Bruker Icon) and white light interferometry (Zygo NexView). To measure the width of unetched silica nanoslit structures, focused ion beam (FIB, Zeiss Auriga) was employed to extract and thin the structures to less than 100 nm, followed by dark-field Scanning Transmission Electron Microscopy (STEM, Hitachi S-5500) to analyze electron diffraction patterns. For fluorescence imaging of Ce:YAG samples, a white light source with a 450–490 nm bandpass filter was used for excitation, and emission was collected through a 510 nm long-pass filter. Mechanical properties of YAG crystals were tested using an SEM equipped with a Hysitron PI 85 PicoIndenter.

## Supplementary information

Supplementary information for Interferometric Scattering for Optical Tomoslicing of Transparent Solids

## Data Availability

The data that support the plots within this paper and other findings of this study are available from the corresponding author on request.
