# Supplementary material for: Interferometric scattering for optical tomoslicing of transparent solids
Source: Light Sci Appl. 2026 Jun 30;15:297. doi: 10.1038/s41377-026-02344-z (PMC13320215; doi:10.1038/s41377-026-02344-z)
Supplement: Supplementary file 1 — Supplementary information for Interferometric Scattering for Optical Tomoslicing of Transparent Solids [file 41377_2026_2344_MOESM1_ESM.docx]

Supplementary information for

Interferometric Scattering for Optical Tomoslicing of Transparent Solids

Yuan Chai^1^, Hong-Hua Fang^1,3^*, Zhen-Ze Li^1^, Tian-Wei Wang^1^, Shao-Feng Liu^1^, Hong-Ren Chen^1^, Shu-Chang Li^2^, Xiao-Yan Li^2^, Jia-Ming Lyu^1^* & Hong-Bo Sun^1^*

**Affiliations:**

^1^State Key Laboratory of Precision Measurement Technology and Instruments, Department of Precision Instrument, Tsinghua University, Beijing 100084, China.

^2^Mechano-X Institute, Applied Mechanics Laboratory, Department of Engineering Mechanics, Tsinghua University, Beijing 100084, China.

^3^Key Laboratory of Advanced Light Conversion Materials and Biophotonics, School of Chemistry and Life Resources, Renmin University of China, Beijing 100872, China

***Corresponding author. Email:**

[hfang@mail.tsinghua.edu.cn](mailto:hfang@mail.tsinghua.edu.cn); [20252758@bistu.edu.cn](mailto:20252758@bistu.edu.cn); [hbsun@tsinghua.edu.cn](mailto:hbsun@tsinghua.edu.cn);

1. **Optical Limitations in Longitudinal Resolution of Laser Processing**

In conventional laser processing, improving longitudinal resolution is much more difficult than enhancing lateral resolution because of fundamental optical constraints. This mainly results from the properties of the focused laser spot and the limitations of focusing optics. A detailed analysis is as follows:

***The Role of Numerical Aperture and Focusing Angle***

The numerical aperture (NA) of an objective lens is a critical parameter in focusing optics. A limited NA restricts the maximum laser focusing angle, denoted as *θ* (NA = *n*·sin*θ*), where *n* is the refractive index of the medium. A smaller focusing angle *θ* due to a limited NA means that there is a significant lack of large-angle transverse wavevectors$k_{\perp}$, which will be less constrained in the longitudinal direction, resulting in an elongated focal spot along the optical axis (Fig. S1a). For optimal longitudinal resolution, a wide range of wavevectors, especially those with large transverse components, is necessary. When these transverse wavevectors are absent, the interference pattern is less constrained in the longitudinal direction, consequently resulting in an elongated focal spot along the optical axis.


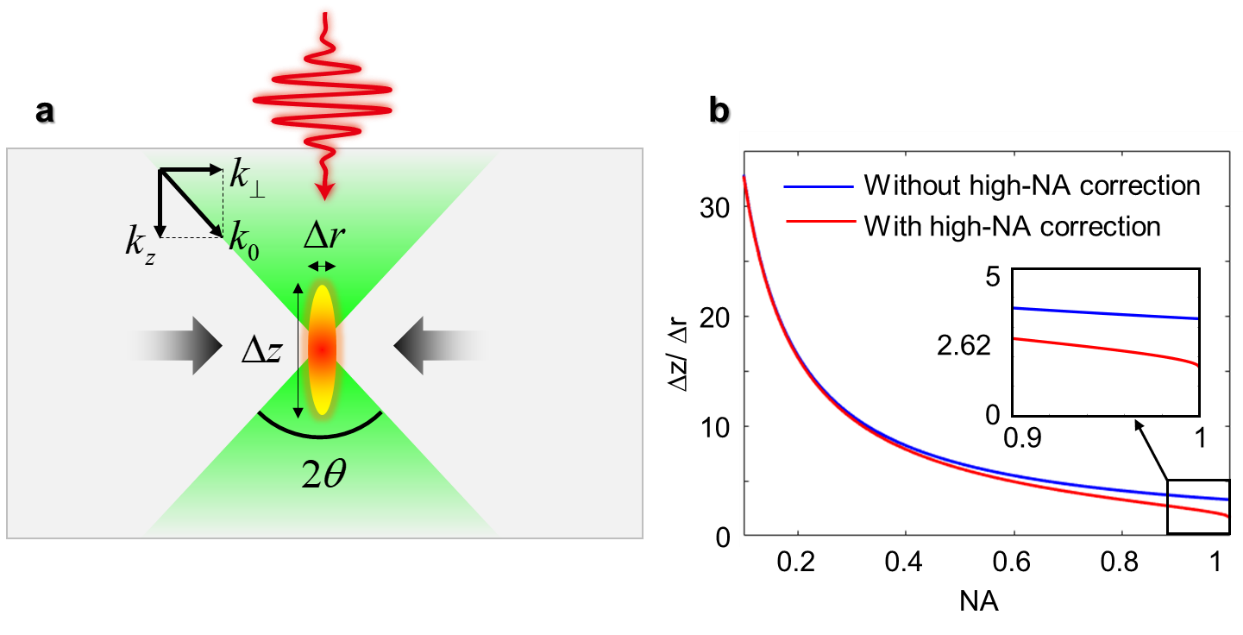


Fig. S1: (a) Comparison of radial and axial wave vector components in optical focusing. (b) The ratio of the longitudinal to transverse dimensions of a focused Gaussian beam in air. The blue line represents the scalar diffraction calculation results, while the red line shows the vector diffraction calculation results after high-NA correction.

***The Intensity Distribution of Focused Light Field***

The focused electric field can be expressed as a vector Debye integral (Richards-Wolf integral) of the field on the objective aperture plane^1^:

 (1)

where $E_{0}$ is the intensity distribution of the incident light field that satisfies a Gaussian distribution. For the low NA (NA<0.7) condition, the spot's radial intensity follows an exponential Gaussian decay, while the axial intensity exhibits a slower Sinc-function attenuation (Fig. S1b), expressed as:

$\left\{\begin{matrix} I(r)=I_{0}\left[\frac{2J_{1}(v)}{v}\right]^{2},v=\frac{2\pi}{\lambda}\cdot NA\cdot r \\ I(z)=I_{0}\left[\frac{\sin(u/4)}{u/4}\right]^{2},u=\frac{2\pi}{\lambda}\cdot\frac{NA^{2}}{n}\cdot z \end{matrix}\right.$ (2)

Therefore, the diffraction limit expression defined by the zero position is:

$\left\{\begin{matrix} \Delta r=\frac{0.61\lambda}{NA} \\ \Delta z=\frac{2\lambda}{NA^{2}} \end{matrix}\right.$ (3)

However, for high-NA (NA>0.7) focusing, higher-order terms must be considered to account for the more stringent intensity distribution. The longitudinal component of the focused field on the optical axis can be expressed as:

$E(0,0,z)=C\int_{0}^{\arcsin(NA)} \sqrt{\cos\theta}e^{ikz\cos\theta}\sin\theta\cos\theta d\theta$ (4)

where *C* is a normalized constant. Therefore, the axial size of the focused spot under high NA satisfies:

$\Delta z=\frac{\lambda}{NA^{2}}(1+\sqrt{1-NA^{2}})$ (5)

Figure S1b shows the ratio of the longitudinal to transverse dimensions of a focused Gaussian beam in air, with the dimensions defined by the positions of the first zero points along the axial and radial directions, respectively. As shown in the figure, the difference between the longitudinal and transverse dimensions gradually decreases with increasing NA. However, even at NA=0.9, the longitudinal dimension remains 2.62 times larger than the transverse one.

Therefore, the longitudinal dimension of the focused laser spot is inherently large, and it is difficult to reduce due to the aforementioned optical limitations, posing a challenge for improving axial resolution in laser processing. This is especially important for applications where a small NA (~0.6) is commonly used for wafering.

1. **Comparison Between Wafer Slicing Methods**

Traditional wafer slicing primarily employs diamond- or slurry-based wire cutting (Fig. S2a). Limited by the diameter of the diamond wire, the kerf loss caused by this method can reach a minimum of 40 μm, while the surface roughness can achieve several-micrometer level. Considering subsequent polishing and grinding steps, for processing 130-μm-thick wafers, the total material loss amounts to 25%.

Laser-assisted wafer slicing technology (Fig. S2b) can process super-hard materials such as SiC and diamond. This method introduces stress by creating modified layers inside the material, then expands the cracks through stress propagation to spall the cap layer. To induce sufficiently large stress, the entire length of the focal spot must be utilized. As a result, the slicing loss of this method is limited by the diffraction limit, typically around 20 μm.

Instead, we propose femtosecond laser interferometric-scattering technology for optical tomoslicing (*i*-SOT) technique, which induces a modificative layer consisting of closely arrayed nanopores by a laser wire sawing tool (Fig. S2c). The longitudinal resolution of *i*-SOT technology is determined by the size of nanopores created by micro-explosion, which is excited by a focused femtosecond laser in transparent solids. The kerf width would be decreased to the nanometer order, for example, a 7-nm width in fused silica, which enables true kerf-free wafering, e.g., with mass loss less than 1%.


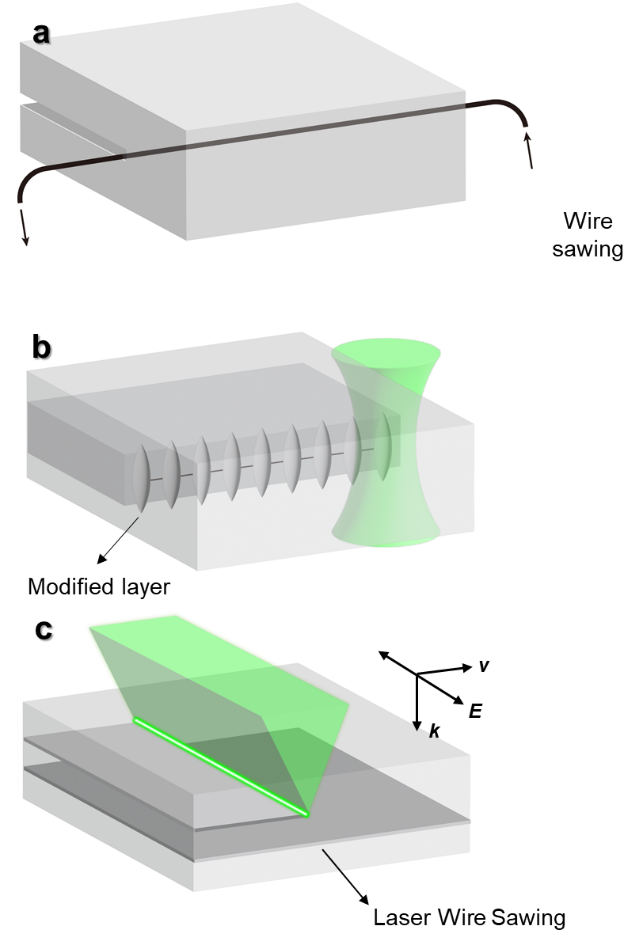


Fig. S2 Comparison among different cutting methods. (a) Traditional wire sawing. (b) Laser-assisted wafer slicing. (c) Interferometric-scattering technology for optical tomoslicing (*i*-SOT) technique.

The table below summarizes the key performance differences between interferometric-scattering technology for optical tomoslicing (*i*-SOT, Our Work) and the current leading cutting techniques. Since 50-μm kerf width has been achieved in industry, while laboratory research has realized kerf widths of several micrometers. Our technology can achieve sub-micrometer kerf width with superior surface smoothness, and this technique can be rapidly adopted in industrial applications.

Table S1: Comparison Between Laser Cutting Methods

| **Ref.** | **Principle** | **Kerf Width** | **Roughness** | **Lab or Industry** |
| --- | --- | --- | --- | --- |
| ^2^ | Laser assisted cold split | ~50 μm | 1.48 μm | Industry |
| ^3^ | Laser assisted cold split | ~50 μm | 0.79/1.29 μm | Industry |
| ^4^ | KABRA | ~80 μm | / | Industry |
| ^5^ | Heat assisted nanocrack generation | >1.5 μm | 89.3 nm | Lab* |
| ^6^ | Laser writing and tensile force exfoliation | ~24 μm | 5 μm | Lab |
| ^7^ | Laser modification and photo-electrochemical exfoliation | ~20 μm | 5 μm | Lab |
| ^8^ | Ps laser micro-explosion and tensile force exfoliation | >2 μm | 1.8 μm | Lab* |
| ^9^ | CW laser assisted and tensile force exfoliation | >2 μm | 1.22 μm | Lab* |
| ^10^ | Diamond wire cutting | 54.2 μm | 0.128 μm | Lab |
| Our work | Interferometric-scattering technology for optical tomoslicing (i-SOT) | < 1 μm | 9/160 nm | Lab/Industry |

* Under laboratory conditions, the damage layer thickness of cracks between the laser-modified zone is relatively small (less than 200 nm), while the actual total thickness of the laser-modified layer and total kerf loss will be larger than cracks.

1. **Simulation of Directionally Controlled Nanoseed Scattering**

The simulation was performed using COMSOL's electromagnetic field model. Figure S3a shows the initial seed and focused laser beam without scattering. In the simulation, we assume that the initial nanopore has a slightly lower refractive index^11^ (∆n=-0.2) than the surrounding material (n_0_=1.46). The simulation uses a laser with a 515 nm wavelength and a numerical aperture (NA) of 0.6. Each new laser pulse is shifted 140 nm to the right of the previous one.

Specifically, the entire model has a height of 5 µm and a width of 2 µm, and the material is silica(utilizing only its refractive index parameter). The radius of the nano-seed is 20 nm, with a refractive index 0.2 lower than that of silica. The physical field is set to "Electromagnetic Waves, Frequency Domain." The waist of the Gaussian beam is defined as w0 = 0.61λ/NA, excited by a Z-direction electric field with Ez = 1 V m-1. The outer boundary is assigned the "Scattering Boundary Condition," and the mesh uses the default "Extremely Fine" setting.

Figure S3b shows the relative electric field intensity distribution when the nanopore is excited. When excited by the laser, the nanopore scatters light, and the relative electric field distribution forms concentric rings centered around the nanopore, creating ~1% intensity perturbations. This electric field distribution is consistent with that of an electric dipole. When excited, the nanopore oscillated as an electric dipole along the direction of the electric field. Since the electric field was perpendicular to the paper plane, the nanopore scattered waves that propagated circularly outward^12^.

Figure S3c illustrates how the light scattered from the nanopore interferes with the subsequent train of waves in the same pulse. This interference creates a series of intensity maxima to the right of the nanopore. The major speckle (0-order) is located directly next to the nanopore and provides an ideal condition for the production of secondary nanopores due to micro-explosion. To provide a clearer view, the right panel zooms in on the interference pattern (with an intensity range of 0.95 to 1.12). Figure S3d presents the relative electric field distribution between two nanopores, revealing a near-circular diffusion pattern centered around both nanopores, which is consistent with Fig. 1c of the main text. The abscissa position of the interference maximum point lies between the previous nanopore and the pulse. Due to the large intensity ratio between the incident beam and the scattered light (close to 100:1), the abscissa of the interference extremum point essentially coincides with the center of the pulse.


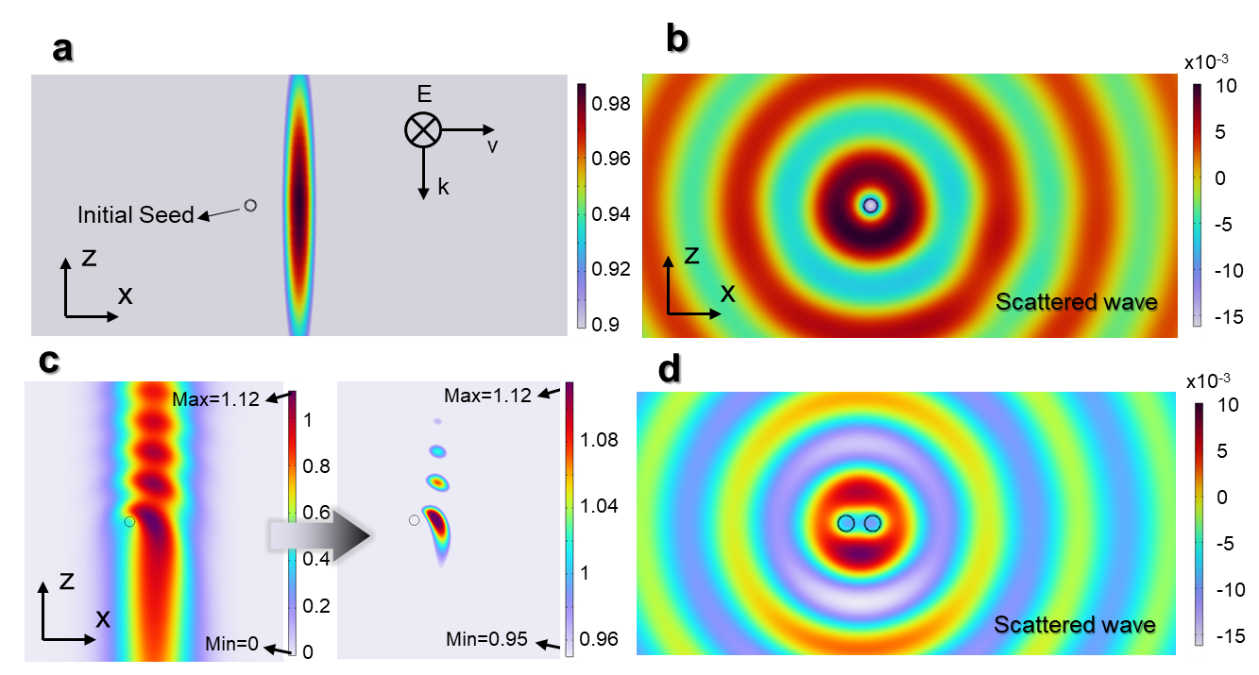


Fig. S3: (a) Laser beam irradiation deviating from the nanopore center. (b) Scattering wave emitted from the excited nanopore. (c) Interference-enhanced distributions at dx_2_=140 nm (Left: the intensity is displayed from 0-1.12; Right: the intensity is displayed from 0.95-1.12). (d) Scattering waves from two excited nanopores.

Figure S4 illustrates the distribution of the coherent field when the distance between the pulse and the end of the nanoslit is 80, 100, and 120 nm. It can be observed that the extreme points of the interference field shift from the upper right to the lower right, while the maximum intensity gradually decreases. These findings are consistent with the conclusions drawn for nanopores.


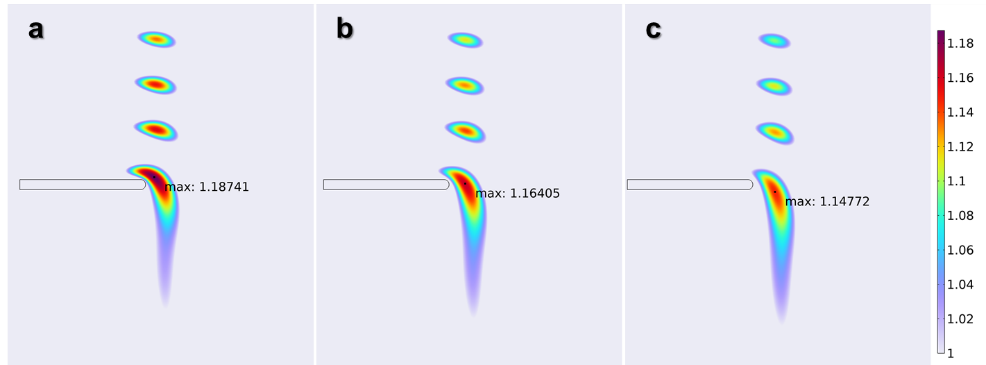


Fig. S4: The distribution of the coherent field when the distance between the pulse and the end of the nanoslit is 80, 100, and 120 nm.

The distribution of the coherent field is insensitive to size and refractive index. In our simulations, the sphere size selected is a radius of 20 nm and a refractive index of 1.25. Figure S5a shows the distribution curve of the interference field intensity along the optical axis for different refractive indices while keeping the sphere radius fixed at 20 nm. It can be observed that the refractive index has almost no effect on the position of the extreme points of the interference field, while a higher refractive index contrast leads to a stronger interference field intensity. Figure S5b shows the distribution curve of the interference field intensity along the optical axis for different sphere radii while keeping the refractive index fixed at 1.25. Similarly, it can be seen that the sphere size has almost no effect on the position of the extreme points of the interference field, whereas a larger sphere size results in a stronger interference field intensity. Since the distribution of the interference field most directly influences the fabrication process, we can conclude that the interference field distribution is robust.


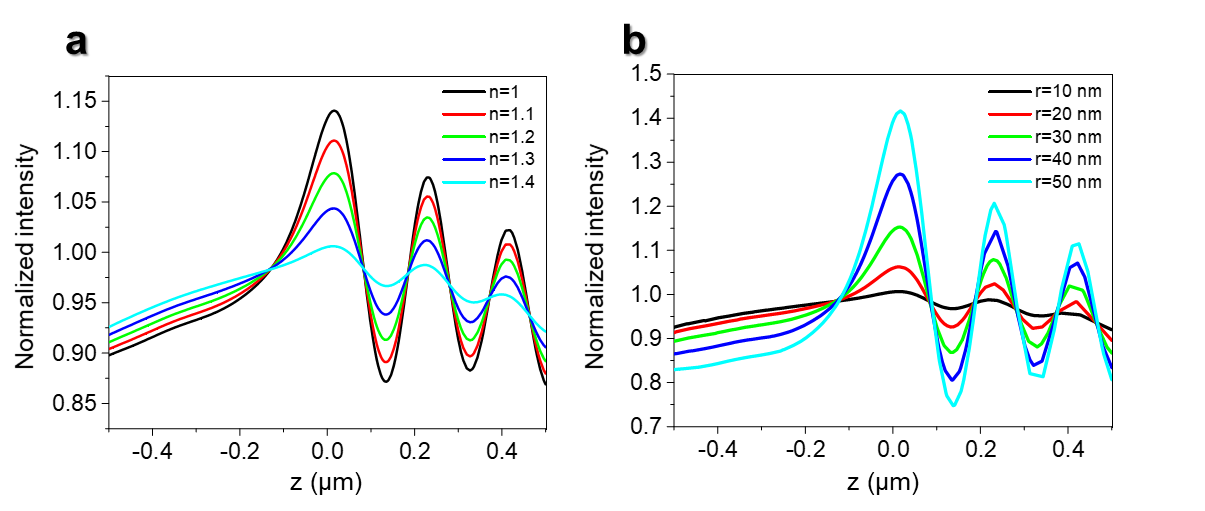


Fig. S5 Simulation results of interference field distribution under different nanopore refractive indexes and sizes. (a) Fixed nanopore radius of 20 nm, electric field intensity distribution along the optical axis under different refractive indexes; (b) Fixed nanopore refractive index of 1.25, electric field intensity distribution along the optical axis under different radii.

Figure S6a presents the electric field intensity distribution in the XY plane when two nanopore structures are irradiated by the optical field, with the electric field polarized along the y-direction. The image reveals that a strong electric field forms in the gap between the nanopores, a phenomenon called "near-field enhancement," which enables connection of the nanopore structures. A distinct fan-shaped light pattern also appears on the right-hand nanopore.

Figure S6b illustrates how these nanopores grow into a nanoslit. We simulated 10 laser pulses, each spaced 120 nm apart. The growth process of the nanopore structures under 10 initial pulses in the XY plane, with a pulse interval of 120 nm and a linear light field polarized along the y-axis. To confirm the simulation, we fabricated these structures deep inside silica and then exposed them by polishing and etching the material. A top-down view shows the nanostructure growing from the nanopores along the y-axis. This growth perfectly matches the fan-shaped field distribution predicted in Figure S6a and stops at the edge of the laser beam, where the energy is too low to continue the process.


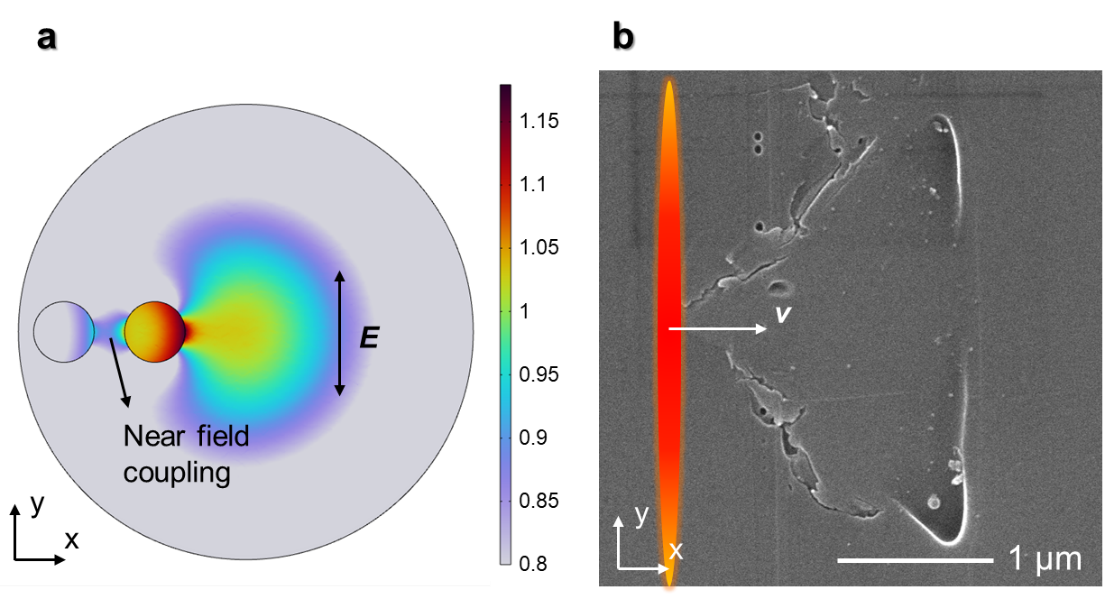


Fig. S6: (a) XY-plane energy distribution after two seeds is irradiated by the optical field. (b) Growth results of seeds in XY planes after irradiation by 10 linear beam pulses with a pulse interval of 120 nm.

1. **The Growth of Tilted Nanoseeds**

Figures S7a-d present the XZ cross-sections of structures formed by 1 to 4 laser pulses, with the beam moving horizontally from left to right in 50 nm steps, that is laser beam offset, δ = 50 nm. As shown in Fig. S7b, with a 50 nm offset between pulses, a secondary nanopore forms at the upper right of the initial one, indicating the direction of tilted growth. Figures S7c-d reveal that as more pulses are applied, the high pulse density induces greater stress, leading to the formation of uncontrolled microcracks around the structures.

After 50 pulses, the resulting uncontrolled microcracks extends beyond the laser's direct scanning path, which is marked by the white rectangle in Figure S7e.

After 100 pulses scanning (Fig. S7f), the growth becomes discontinuous. As the structure extends too far from the laser's center, the scattered field's energy is too weak to keep the nanopore formation in the tilted direction. A new nanopore again forms at the main laser spot.

This behavior is supported by the simulation of the optical field. Figure S7g and h present the simulated electric field that corresponds to these two scenarios. When the laser spot center is offset from the nanoslit (500 nm offset, Figure S7e, g), the nanoslit induces a redistribution of the laser intensity, shifting the peak energy to the upper right and promoting preferential growth in that direction. In contrast, when the laser spot is positioned far from the nanoslit (900 nm offset, Fig. S7h), the peak intensity remains localized at the center of the laser spot. This leads to discontinuous growth, as a new, separate structure is formed.


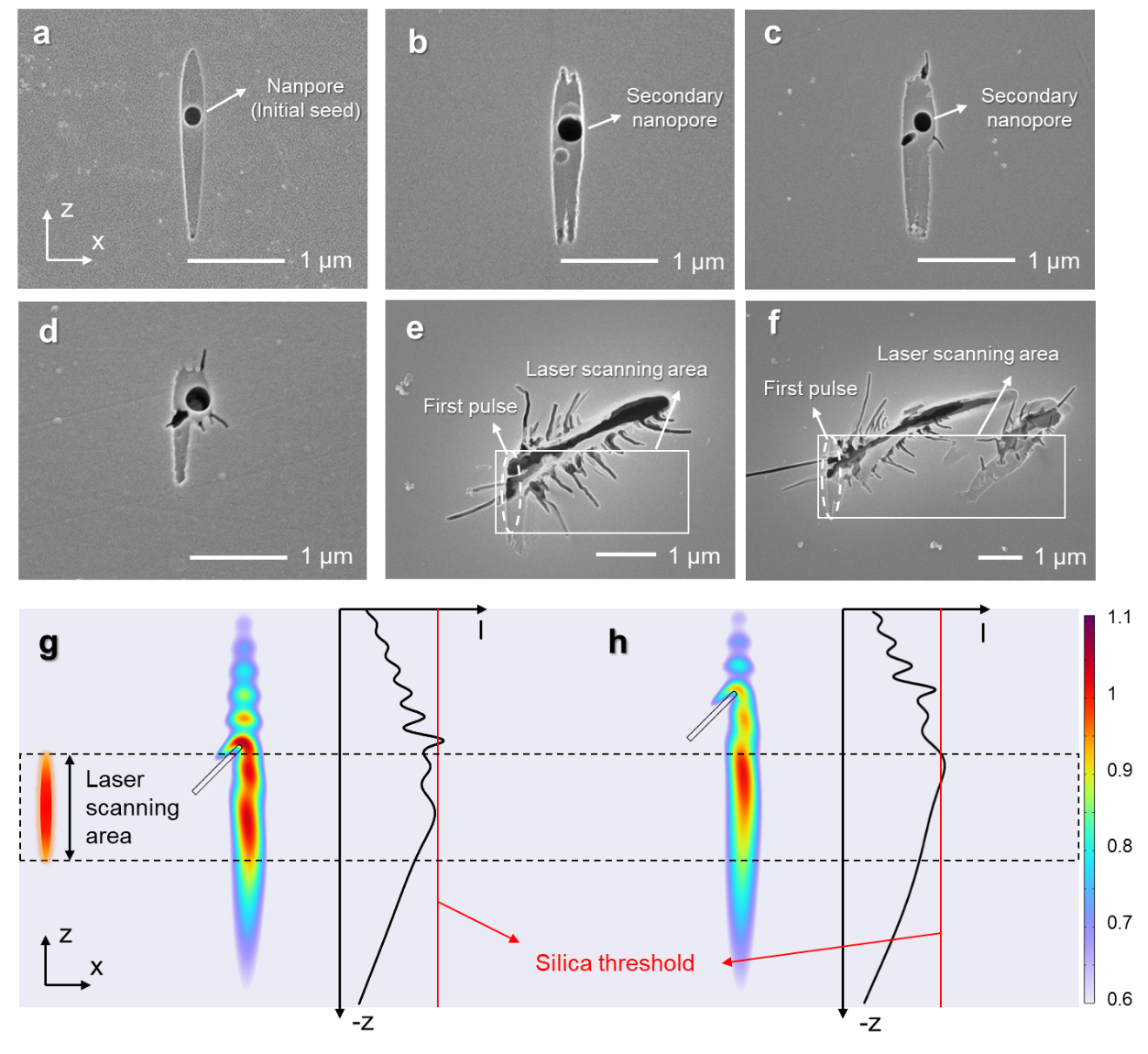


Fig. S7: (a-f) Tilted growth of nanostructure under pulse numbers of 1, 2, 3, 4, 50, and 100, with a 50 nm pulse offset. (g-h) Simulation of the electric field distribution when the nanoslit is offset from the spot center at different distances. Figure g shows a 500-nm offset, while Figure h presents a 900-nm offset from the center.

1. **Structure Evolution Under Different Pulse Intervals**

The pulse interval has a significant influence on the morphology of the structure. As an extension of Fig. 1k in the main text, we analyzed cross-sectional profiles of nanograting/nanoslit structures with laser beam offset, δ ranging from 10 to 180 nm. Figure S8 displays the cross-sectional morphology of structures created with δ ranging from 10 nm to 180 nm. In this setup, the laser scans from left to right, and its electric field is perpendicular to the page.

The orientation of the resulting structure is highly dependent on the δ. We measured the angle of the structure relative to the laser's scanning direction and observed a clear trend:

At a laser beam offset, δ = 10 nm, the structure is tilted upward at 71°.

At δ = 120 nm, it becomes nearly horizontal.

At δ = 180 nm, the tilt reverses to a downward angle of -22°.

Overall, as the pulse offset increases, the tilt direction transitions from the upper-right to the lower-right. The type of structure also changes with the pulse offset: For δ <50 nm, the cross-sectional morphology shows inclined nano-plane structures, representing the side view of Type-II nanogratings. This is because that at smaller offsets, the energy density is sufficiently high to allow nanopore structures to fully develop into nanogratings, as previously reported^13^. When the δ = 60 nm, the reduced energy density predominantly leads to nanoslit formations. Specifically, at δ = 120 nm, these nanoslits grow horizontally in a stable manner. Unstable, transitional structures are observed in the δ = 60-100 nm and 150-170 nm ranges. This is because the interaction between a pre-existing structure and the next laser pulse weakens, interrupting continuous growth, as discussed in Figure S7. At the pulse offset δ = 190 nm, the pulses are too far apart to interact, leading to the development of entirely separate, isolated structures.


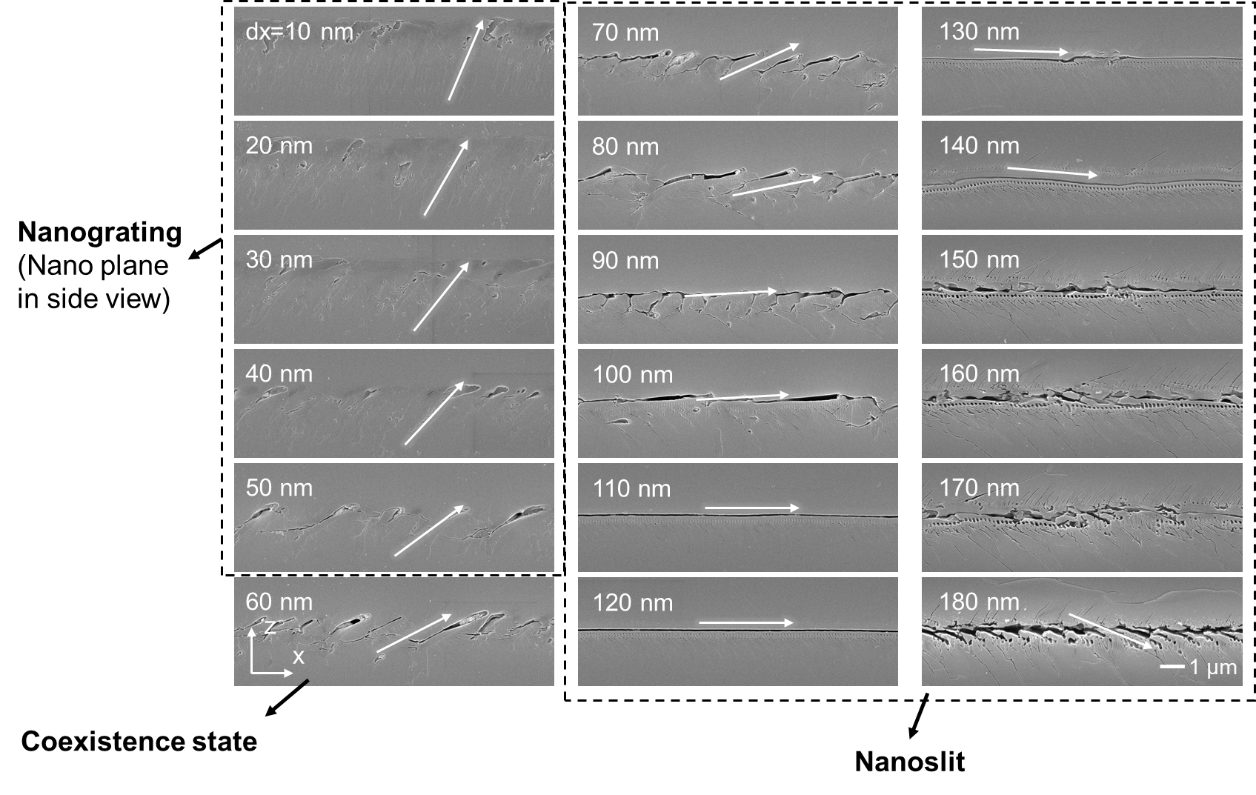


Fig. S8: Comparison of tilt angles under different pulse offsets

Figure S9(a-b) illustrate how the spacing between laser pulses determines the final structure. the schematic diagrams of nanograting and nanoslit structures fabricated with δ = 20 nm and 120 nm, respectively. Figure S9(c-h) provides a comparative analysis of the cross-sectional morphologies fabricated using a line optical field with a width of 50 μm, specifically along the scanning direction (Fig. S9(c-e)) and perpendicular to the scanning direction (Fig. S9(f-h)), with δ = 20 nm (Fig. S9c and S9f), 80 nm (Fig. S9d and S9g), and 120 nm (Fig. S9e and S9h), respectively. The results show that at a high pulse density (50 pulses per μm), that is, a short pulse offset (δ = 20 nm), the cross-sections exhibit tilted nanoplane structures (Fig. S9c) along the scanning direction and nanograting structures (Fig. S9f) in the perpendicular direction. When the δ = 120nm, both directions display nanoslit structures. While δ = 80nm, unstable nanoslit structures are observed.


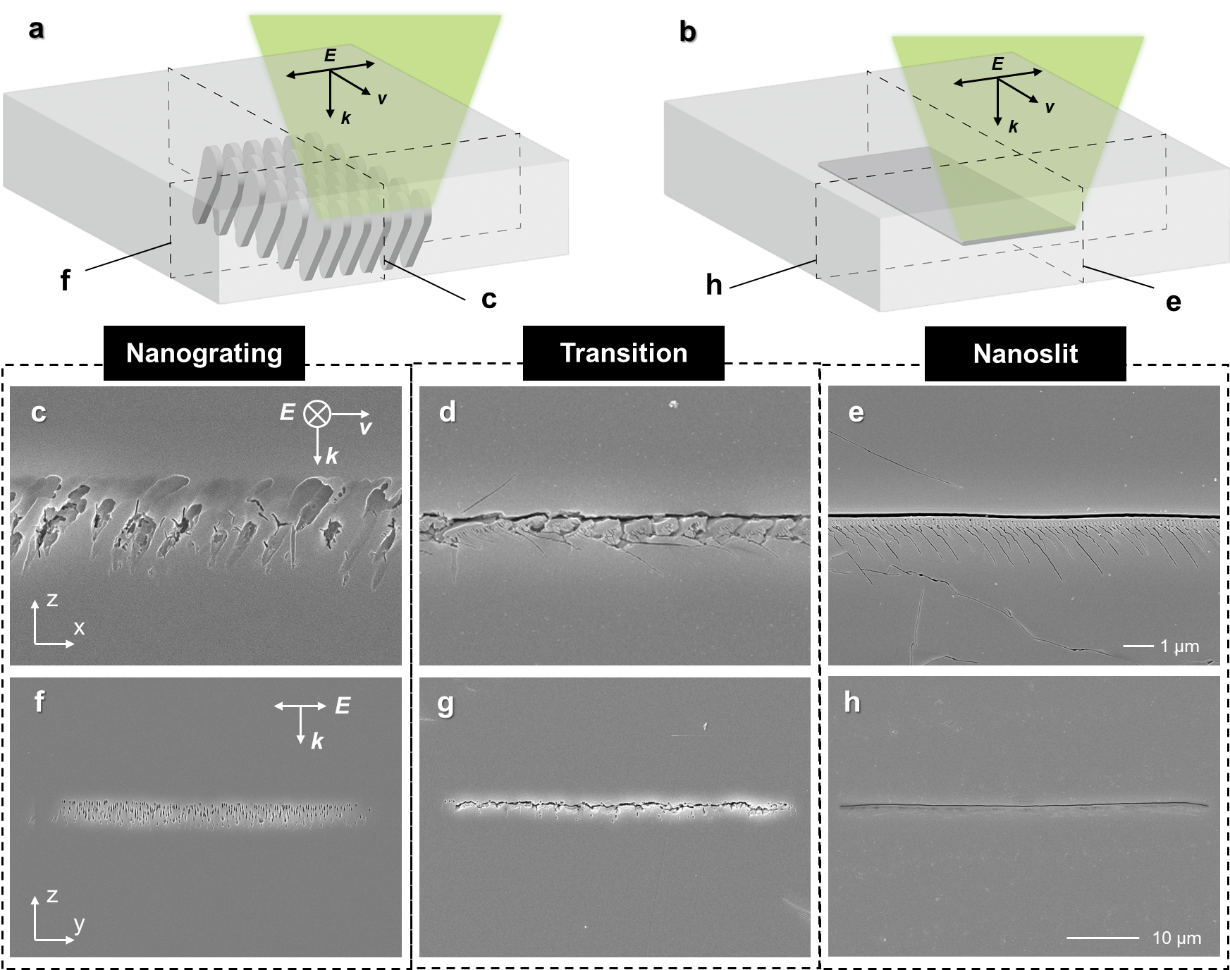


Fig. S9: (a-b) Schematic diagrams of nanogratings and nanoslits fabricated with pulse offsets of 20 nm and 120 nm. (c-e) Cross-sectional morphologies along the scanning direction and (f-h) perpendicular to the scanning direction. The pulse offsets are 20 nm, 80 nm, and 120 nm, respectively.

Figure S10 shows the birefringence changes under different pulse spacings to further characterize the evolution of nanograting and nanoslit structures. Here, color represents changes in birefringence angle, while brightness and darkness reflect the magnitude of birefringence retardation. As shown in the figure below, as the pulse spacing increases, the birefringence color shifts from red to green, and the birefringence angle rotates by 90 degrees. This is because the birefringence property of nanogratings is structural birefringence, with the optical axis parallel to the electric field direction (perpendicular to the nanoplanes), whereas nanoslit structures are dominated by stress birefringence, aligned with the scanning direction. The changes in birefringence also illustrate, from another perspective, the evolution from nanogratings to nanoslits.


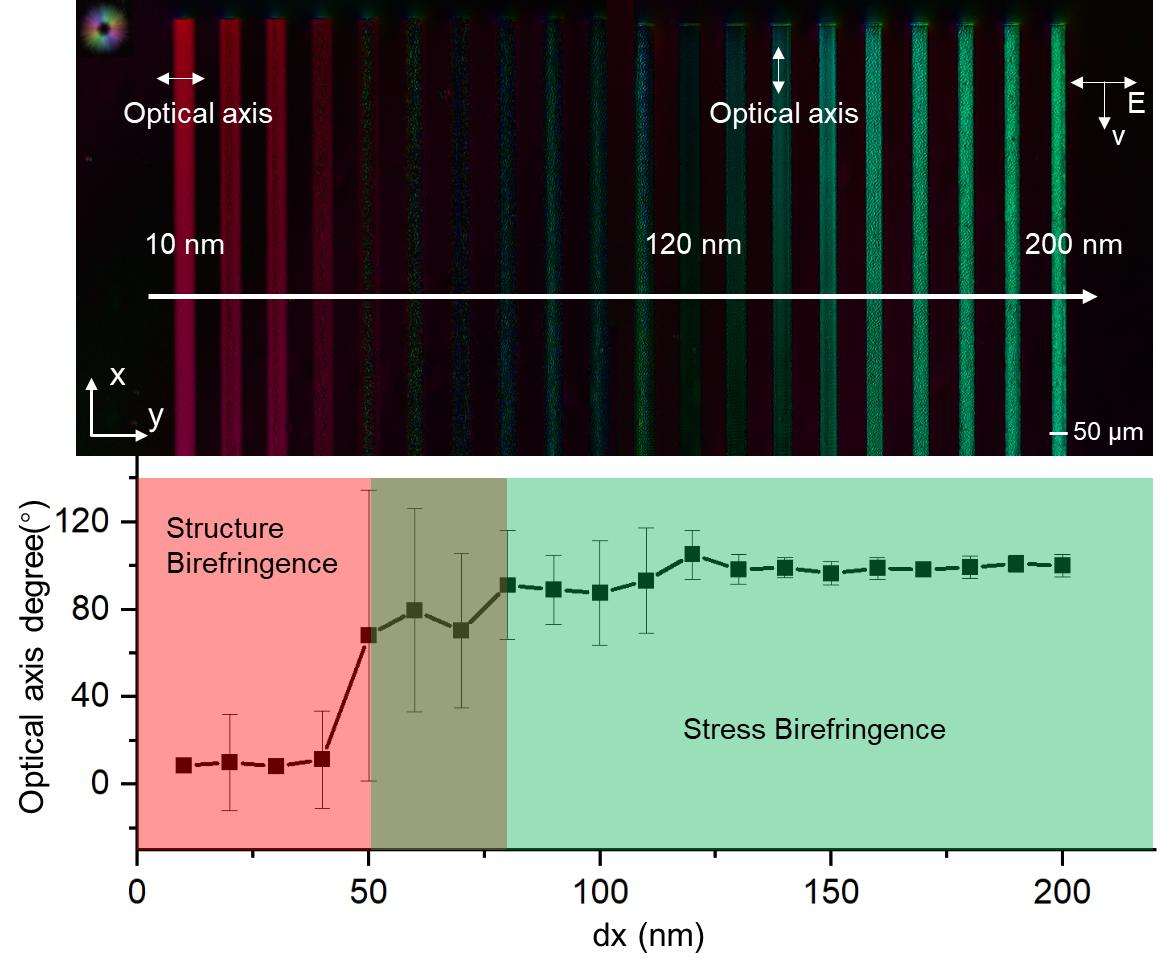


Fig. S10 The birefringence angle varies with the pulse interval.

1. **Operational windows for nanoslits’ procession**

In the experiment, we employed an objective lens with NA 0.6 to focus a 515 nm, 230 fs laser into a 50 μm long line spot, with a single pulse energy of 3.3 μJ. For energy control, we needed to meet the near-threshold condition, which helps avoid the sub-maximum points in the interference field exceeding the threshold and thus causing damage to the nanoslits structure.

From Fig. 2a, it can be seen that for the NA0.6 objective lens, the tilt angle range of the nanogrooves is α = -10° to 30°. To achieve nanoslits with larger angles (α > 30°), a smaller pulse interval (δ < 60 nm) is required, which would lead to further growth of nanoseeds into nanogratings, thereby losing the sub-diffraction-limit characteristic of the nanoslits. Conversely, to achieve nanoslits with smaller angles (α < -10°), a larger pulse interval (δ > 150 nm) is needed. In this case, the intensity difference between the maximum and sub-maximum points in the interference field is very small. Considering the threshold effect in laser processing, it is difficult to stably generate the nanoslit structure.

In addition, we tested the processing effect under the NA 0.95 objective lens. Due to the smaller spot size of the NA 0.95 objective, the axial attenuation of its interference field intensity is faster, and the pulse interval range suitable for processing nanoslits is shorter. This is because at shorter intervals, multi-pulse accumulation is more likely to grow into nanogratings, while at longer intervals, pulses do not affect each other. We also attempted processing with an NA0.4 objective lens in experiments and found that its spot could hardly form stable nanoslit structures. The main reason is that at low NA, the intensity difference between sub-maximum and main maximum points is too small, making it easy to exceed the threshold and cause structural disorder.

1. **The crack structure of the nanoslit and its annealing healing effect.**

The intensity of stress birefringence in the nanostructure reveals significant structural stress within it. Upon closer inspection, comb-like cracks can be observed beneath the nanostructure, oriented at an angle of approximately 48° relative to the direction of the nanostructure. When the laser is focused inside the material to induce micro-explosions, rapid cooling and solidification inevitably generate substantial internal stress that must be released. In the fabrication of nanogratings, despite the higher pulse density used during processing—which theoretically should result in greater stress—the alternating dense and sparse structural characteristics allow the stress to be fully released through the material's varying density.

In contrast, for the nanoslit, the intense internal stress is concentrated within an extremely thin longitudinal modified layer, leading to stress release primarily in the form of cracks. The overall alignment of the crack orientation with the scanning direction (both oriented rightward in the image below) further confirms that crack propagation progresses from the modified high-stress region (left side) to the unmodified low-stress region (right side). These cracks manifest as atomic-scale separations, with the cracks shown in the image below being the result of etching for amplification. If the nanoslit were directly used in subsequent processing, these cracks would significantly reduce the surface flatness of the structure, severely compromising processing quality. Fortunately, the extent of damage caused by these cracks is far less than that of the nanoslit itself, resulting in a lower tolerance to high temperatures compared to the nanoslit, and a lower healing temperature for the cracks than for the nanoslit. We performed annealing in a high-temperature environment above the healing temperature of the cracks but below that of the nanoslit, with an annealing temperature range of 1050–1100°C in the experiments. Under thermal motion, atoms on both sides of the cracks rebonded. As shown in the image below, not only were the cracks completely healed after annealing, but the Type-I weak modifications also entirely disappeared, leaving only the nanoslit intact.


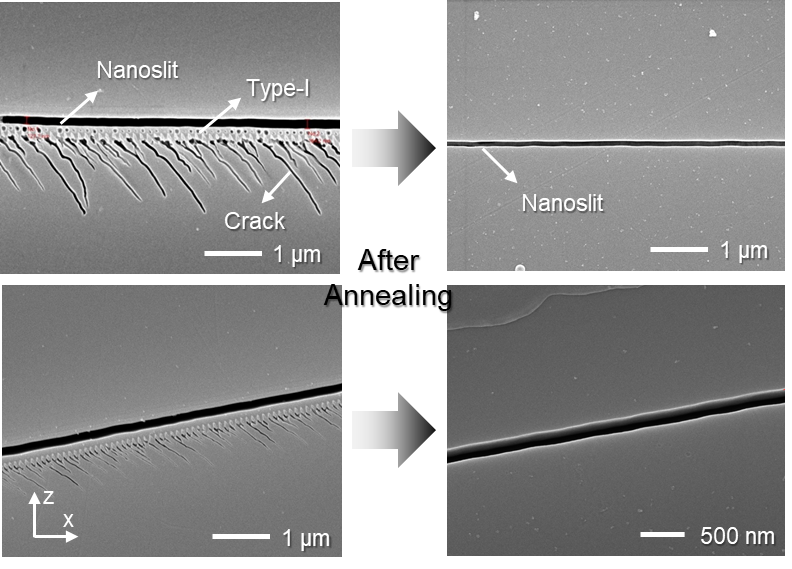


Fig. S11 The crack structure of the nanoslit and its annealing healing effect.

1. **Splicing Effect on Slicing Roughness**

When the laser linear light field is shorter than the wafer, it needs to scan and splice multiple regions together to process large areas. Figure S12b illustrates the scanning and splicing method of the line optical field within the XY plane. A single scan with the line optical field can fabricate nanoslit structures with approximately 40 μm wide. To ensure continuity, the next scan must be placed at a distance of less than 40 μm, matching the silica damage zone. However, the already-formed nanoslit absorbs light more effectively than the surrounding material, resulting in a lower damage threshold compared to the unprocessed region (Fig. S12a). This causes a 5-µm wide over-processed region at the splice point (Fig. S12b), which reduces the overall flatness of the sliced surface.


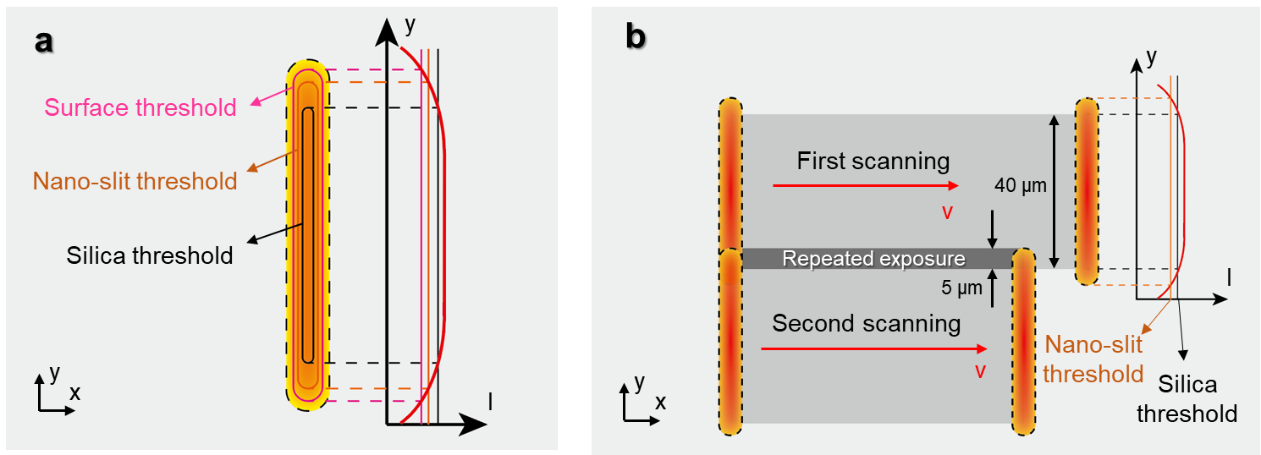


Fig. S12: (a) Relative ranges of different damage thresholds of the line light spot in the XY plane. (b) Schematic of a nanoslit double exposure caused by line spot stitching.

To reduce the impact at the stitching points, the stitching width can be reduced by modulating a line spot with steeper edge intensity, while the overall uniformity can be improved by increasing the length of the line spot.

In industrial applications, wafers often require sub-nm-level flatness. Therefore, although there are no stitching seams, subsequent polishing is still necessary. However, our slicing technology only requires a certain degree of fine polishing, and the total slicing loss can still be controlled at the sub-micron level.

1. **The Influence of Longitudinal Light Field Distribution on Adjacent Slicing Distance**

The laser's light field distribution determines the necessary spacing for multi-layer and near-surface processing. For multi-layer slicing (Fig. S13b), a bottom-up processing approach is typically adopted to avoid the influence of pre-existing nanoslit structures on light field propagation. The interlayer distance ∆z_1_ must exceed the focal spot range corresponding to the nanoslit damage threshold (Fig. S13b) to avoid reablation of the pre-existing slicing plane. In experiments, we tested this distance using an NA 0.6 objective lens, and found that this interlayer distance ∆z_1_ should be > 2.2 μm.

Similarly, for near-surface slicing (Fig. S13c), the distance from the structure to the surface (Δz_2_) must be carefully controlled. Compared to nanoslit structure, the surface is more susceptible to damage due to two primary factors: (1) direct exposure to air makes surface material more prone to splattering, whereas internal processing benefits from material confinement provided by the surrounding lattice; and (2) surface contamination and surface defects enhances photon absorption, leading to a significantly lower damage threshold than the bulk material's modification threshold. Therefore, the processing spot must be kept far enough away to avoid damaging the surface. Experimentally, we found this distance must be greater than 6 µm, when using an NA 0.6 objective lens.


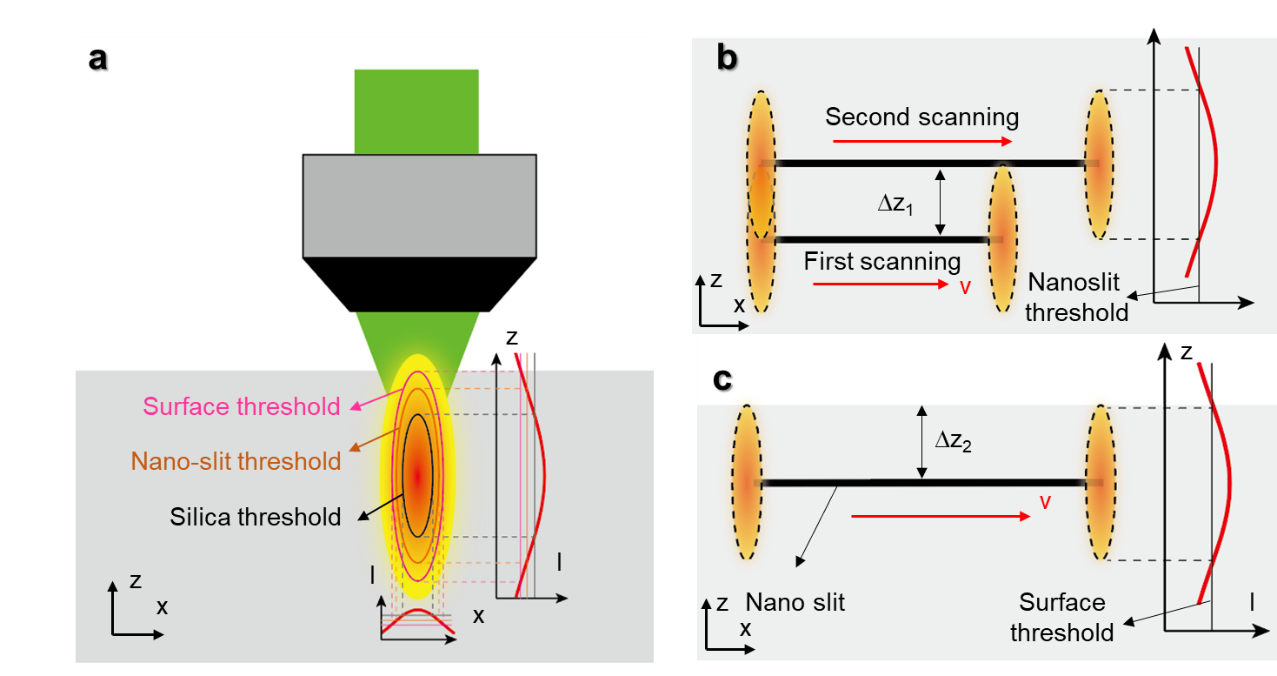


Fig. S13: (a) Relative ranges of different damage thresholds of the line light spot in the XZ plane. (b) Schematic of the minimum interlayer spacing for multilayer slicing. (c) Schematic of the nearest cutting distance for subsurface slicing.

1. **The Growth of Nanostructure in YAG and MgAl2O4 Crystals**

The optical field redistribution that guides nanostructure growth is not unique to fused silica; it also occurs in crystalline materials. Figure S14 shows cross-sectional variations of modified structures in YAG and MgAl_2_O_4_ under different pulse offsets (the pulse interval is 60-200 nm for YAG and 10-170 nm for MgAl_2_O_4_). It can be observed that as the pulse interval increases, the seed's tilt direction gradually approaches the horizontal. This horizontal growth was achieved at a pulse interval of 150 nm for YAG and 120-150 nm for MgAl₂O₄, demonstrating that the principle of scattering interference effect is broadly applicable to different materials.


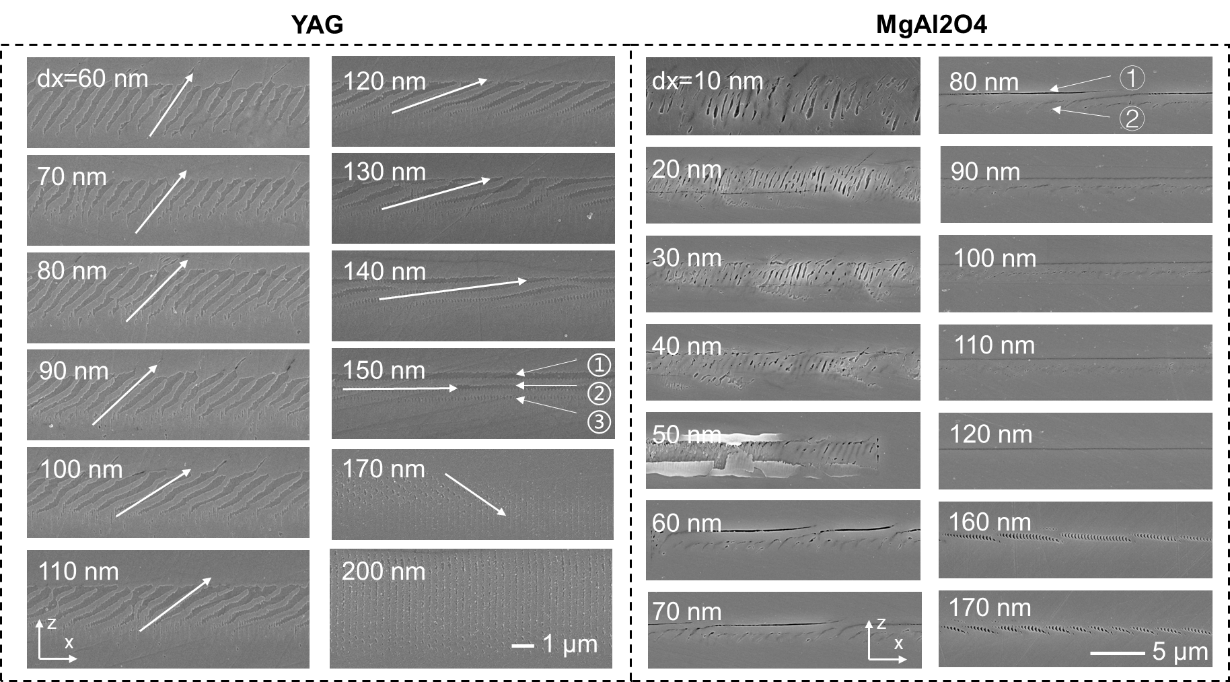


Fig. S14: The stripe inclination directions of YAG crystals and MgAl_2_O_4_ crystals vary with the pulse intervals.

Figure S15 displays the nanopore structures fabricated inside MgAl₂O₄ crystal at different pulse energies, with a pulse interval of 140 nm. When the energy of a single pulse is 4.66 μJ near the damage threshold of MgAl₂O₄, the structures consist of isolated nanopore arrays (Fig. S15a). When the energy is increased to 5.48 μJ, the nanopores become laterally interconnected due to micro-explosions, forming nanoslits with a longitudinal dimension of only 70 nm (Fig. S15b). This structure can be utilized for subsequent crystal slicing.


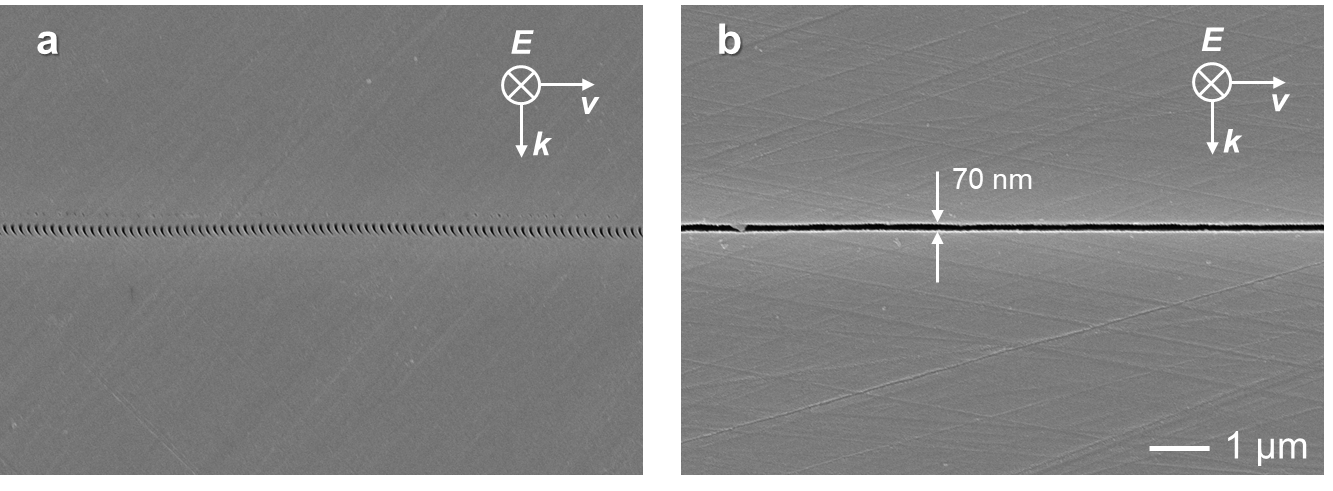


Fig. S15: Nanostructure fabricated inside MgAl₂O₄ crystal with a pulse interval of 140 nm. The energy of each single pulse is 4.66 μJ and 5.48 μJ, respectively.

However, the formation of nanostructures in crystalline materials is different from that in fused silica. Figure S16(a-b) presents the nanopore morphologies of YAG and MgAl_2_O_4_ at pulse counts of 1, 2, 3, 4, 5, and 10 with a 70 nm pulse offset. As can be seen from the figures, under one pulse irritated, the nanoseed is distributed in a slit-shaped pattern. We believe this is due to the anisotropic nature of crystals, where ionized electrons diffuse preferentially along specific crystal orientations rather than in all directions. Under 10 pulses, the seed morphology exhibits a multi-layered structure, which corresponds to the multilayer structure shown in Fig. S16.

Simulations support these findings (Figure S16(c-d)). The simulation of optical field distributions around slit-shaped seeds was performed with pulse offsets of 70 nm and 130 nm, respectively. The slits measure 20 nm in width and 500 nm in length, with a refractive index of 1.5 (compared to 1.7 for the surrounding material). The results demonstrate that due to the elongated slit along the optical axis, its scattered field coherently interacts with subsequent pulse trains, generating multiple intensity peaks that exceed the damage threshold. This specific field distribution allows for the fabrication of multi-layered structures.


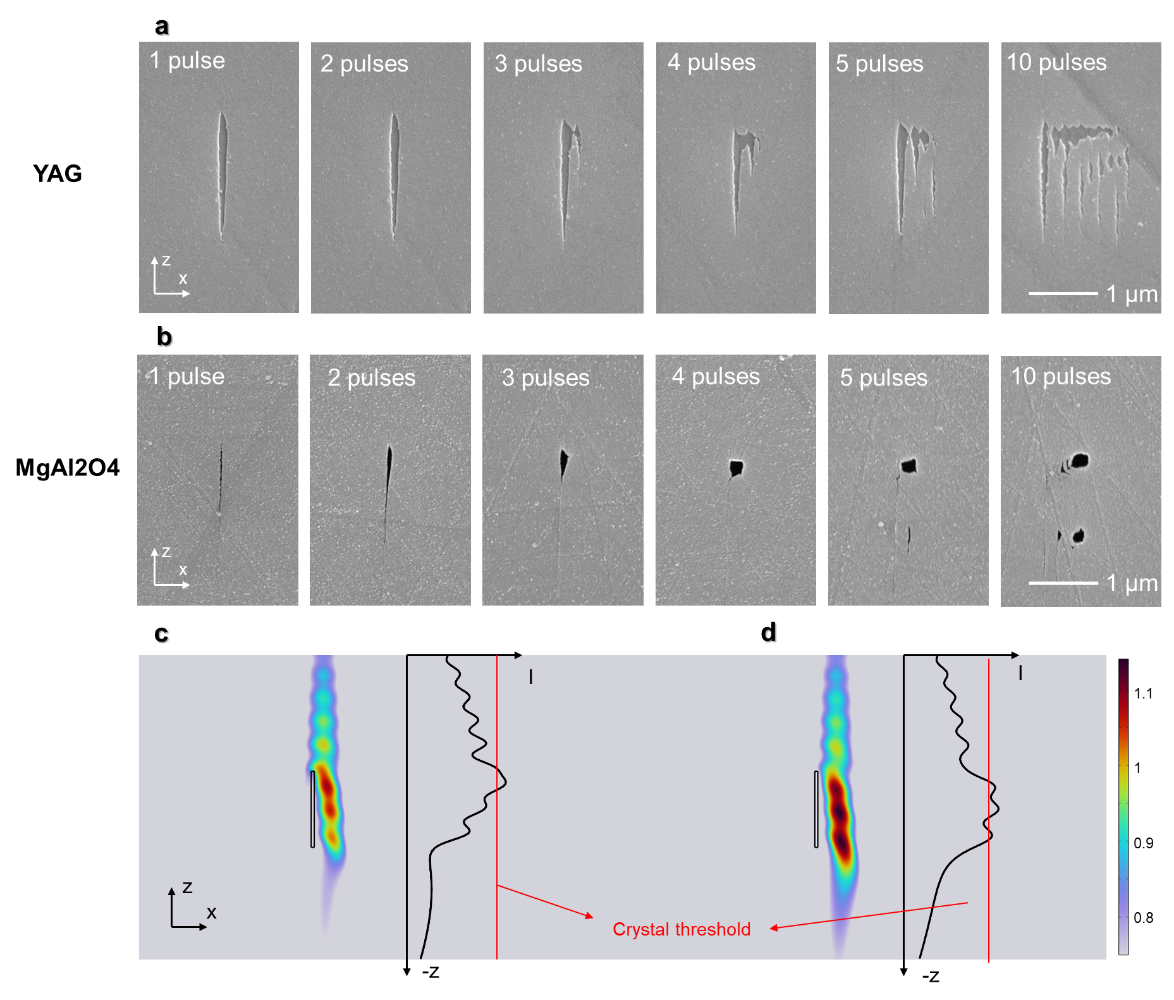


Fig. S16: (a-b) Nanopore morphologies of YAG and MgAl2O4 at pulse counts of 1, 2, 3, 4, 5, and 10 with 70 nm pulse intervals. (c-d) Simulation results of optical field distributions around slit-shaped seeds with pulse offsets of 70 nm and 130 nm.

1. **Slicing and Characterization of YAG and MgAl2O4 Crystals**

To characterize the internal structure of multi-layer sections of the YAG crystal, we performed 3D X-ray imaging. Figure S17d shows the X-ray scan results of five-layer nanoslits, where five dark nanoslits are clearly visible, confirming complete internal separation after etching.

Figure S17b displays the cross-section of a YAG disk section with a thickness of 6 μm. Figures S17(e-f) present the EDS elemental mapping results of the multi-layer YAG slices, corresponding to Figure S17c, where the black lines in the EDS images correspond to the nanoslits between slices.


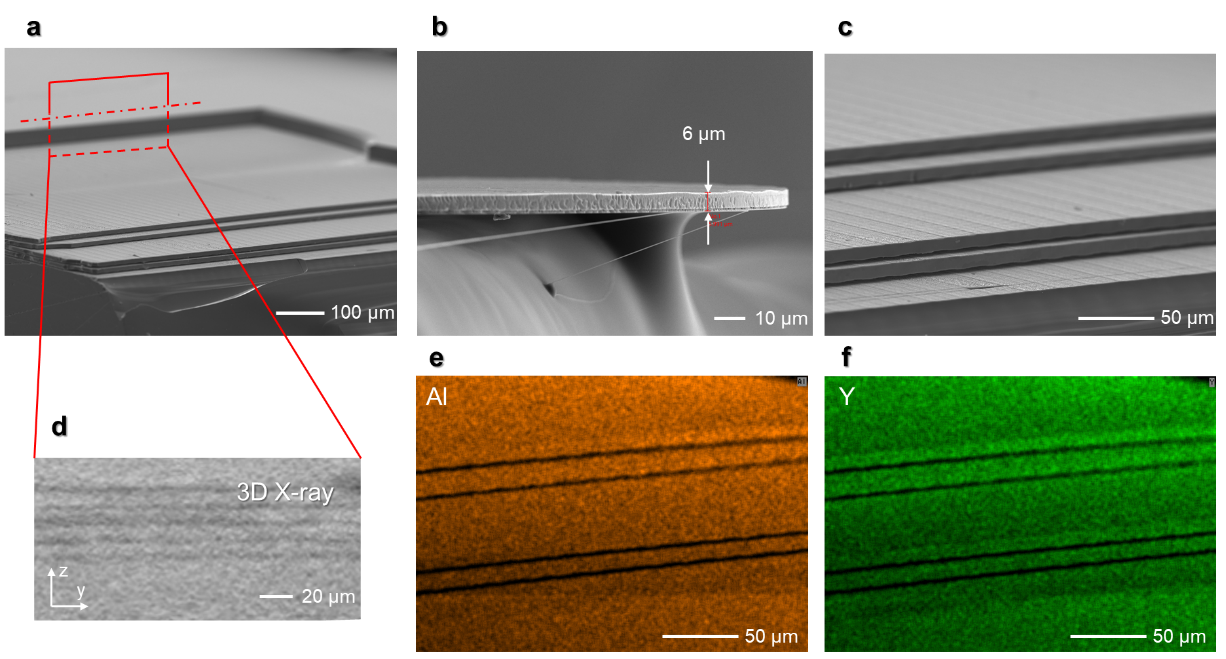


Fig. S17: Characterization of YAG crystal slices. (a) Multilayer YAG slices. (b) Side view of YAG disk slices. (c) Demonstration of multilayer YAG slices. (d) X-ray scanning results of multilayer YAG slices. (e-f) EDS scanning results of multilayer YAG slices, showing Al and Y element distributions respectively

We demonstrated the technique's versatility by creating arrays of disks with various shapes and sizes. Figure S18(a-c) demonstrates array processing of YAG disk slices with a 500-μm diameter and 6 μm thickness. By combining linear light field slicing and single-point contour scanning processing, various shapes of slicing arrays can be fabricated.

Figure S18(d-g) shows white light interference images of differently shaped and sized slicing substrates. Figure S18d displays square substrates with side lengths of 20 μm and 50 μm, both with a depth of 9.2 μm. Figures S18e,f present circular substrates with diameters of 20 μm and 100 μm, each having a depth of 6 μm. Figure S18g illustrates triangular substrates with a side length of 20 μm and a depth of 5.5 μm.

Figure S18i presents a stacked configuration of four disks, where the bottom two slices are placed with their flat surfaces facing upward and the top two with their slit ends facing upward.

Figure S18j shows AFM characterization was performed, revealing a surface roughness of Ra = 13.4 nm within a 10 × 10 μm^2^ area.


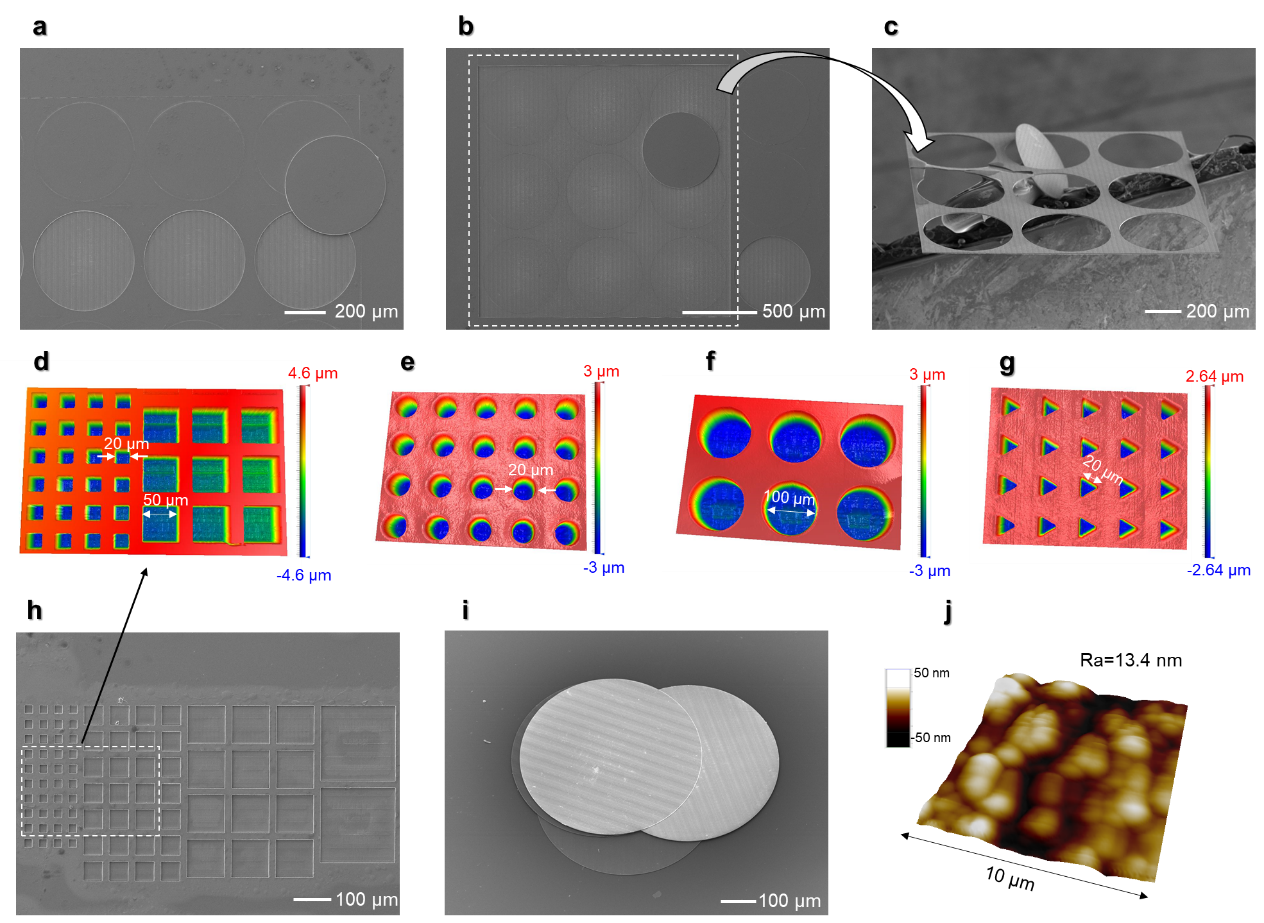


Fig. S18: (a-c) Processing of YAG thin sheet arrays. (d-g) White light interference effects of disc-shaped, square, and triangular YAG thin sheet array substrates of different sizes. (h) YAG square thin sheet array substrate. (i) Stacking effect of YAG circular thin slices (j) Surface roughness of YAG slices

1. **Mechanical Property Characterization of YAG Wafers**

Figure S19(a-f) show the bending effects of the cantilever at different displacements: 0, 80, 160, 240, 320, and 400 μm. The indenter has a diameter of 20 μm, while the cantilever beam is 30 μm wide, approximating an ideal cantilever structure. The cantilever length is 780 μm and thickness is 9.55 μm. The moment of inertia of the cantilever satisfies^14^:

$I=\frac{1}{12}bh^{3}=23.875 \mu m^{3}$ (4)

According to the literature^15^, the Young's modulus of YAG crystal is 308 GPa. Based on this value, the pressure-to-displacement ratio of the cantilever beam can be calculated as^14^:

$\frac{dF}{dy_{B}}=\frac{3EI}{l^{3}}=4.24 N/m$ (5)

The experimentally measured slope of the pressure-displacement curve was 4.42 N m-1, which is well aligned with the theoretical calculated results.

The cantilever shows very high flexibility, as shown in Figure 4h, with a minimum radius of curvature of:

$R_{\min}=\frac{L^{2}}{3y_{B}}=271 \mu m$ (6)


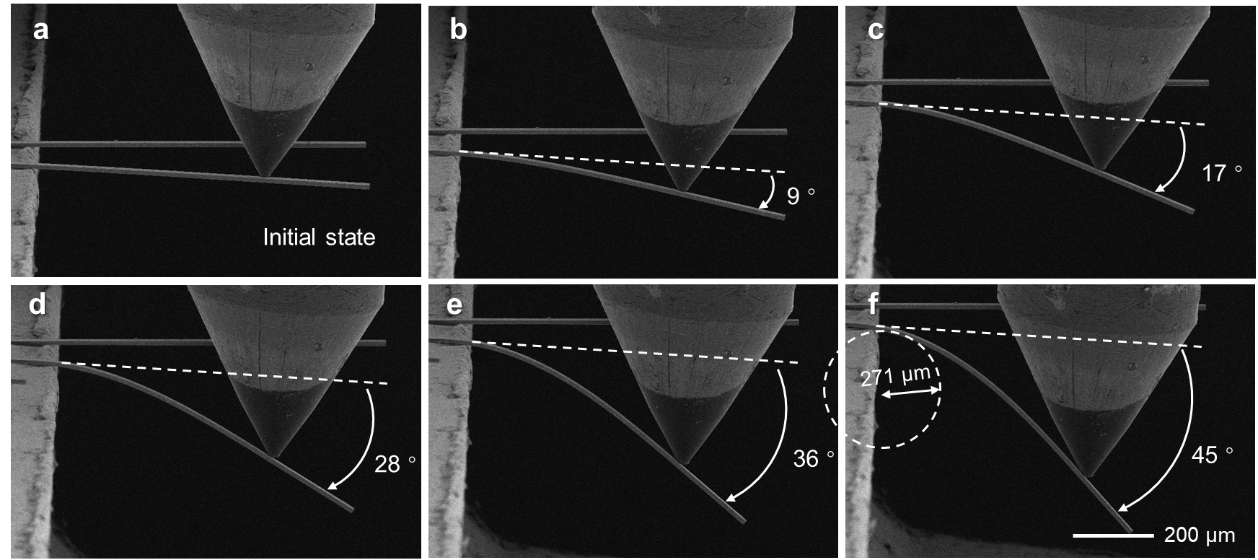


Fig. S19: The bending degree of the YAG cantilever beam structure when the indenter is pressed down by 0,80,160,240,320, and 400 μm, respectively.

References

1 Hu, Y. *et al.* Efficient full-path optical calculation of scalar and vector diffraction using the Bluestein method. *Light: Science & Applications* **9**, 119, doi:10.1038/s41377-020-00362-z (2020).

2 Swoboda, M., Beyer, C., Rieske, R., Drescher, W. & Richter, J. Laser Assisted SiC Wafering Using COLD SPLIT. *Materials Science Forum* **897**, 403-406, doi:10.4028/<www.scientific.net/MSF.897.403> (2017).

3 Kaule, F. *et al.* Laser-assisted spalling of large-area semiconductor and solid state substrates. *MRS Communications* **8**, 127-131, doi:10.1557/mrc.2017.136 (2018).

4 Kling, R., Washio, K., Klotzbach, U. & Hirata, K. in *Laser-based Micro- and Nanoprocessing XII* (2018).

5 Xiangfu, L. & Minghui, H. Heat-assisted pulsed laser processing induced nano-cracks for low kerf-loss and high-surface quality SiC wafer slicing. *Optics & Laser Technology* **189**, doi:10.1016/j.optlastec.2025.113040 (2025).

6 Kim, E., Shimotsuma, Y., Sakakura, M. & Miura, K. 4H-SiC wafer slicing by using femtosecond laser double-pulses. *Optical Materials Express* **7**, doi:10.1364/ome.7.002450 (2017).

7 Geng, W. *et al.* Slicing of 4H‐SiC Wafers Combining Ultrafast Laser Irradiation and Bandgap‐Selective Photo‐Electrochemical Exfoliation. *Advanced Materials Interfaces* **10**, doi:10.1002/admi.202300200 (2023).

8 Han, S. *et al.* Laser slicing of 4H-SiC wafers based on picosecond laser-induced micro-explosion via multiphoton processes. *Optics & Laser Technology* **154**, doi:10.1016/j.optlastec.2022.108323 (2022).

9 Jiang, L. *et al.* CW laser-assisted splitting of SiC wafer based on modified layer by picosecond laser. *Optics & Laser Technology* **174**, doi:10.1016/j.optlastec.2024.110700 (2024).

10 Sefene, E. M., Chen, C.-C. A. & Wang, S. H.-M. Determination of sawing temperature in multi-diamond wire sawing of mono-crystalline silicon carbide. *CIRP Journal of Manufacturing Science and Technology* **60**, 38-55, doi:10.1016/j.cirpj.2025.04.004 (2025).

11 Lancry, M. *et al.* Ultrafast nanoporous silica formation driven by femtosecond laser irradiation. *Laser & Photonics Reviews* **7**, 953-962, doi:10.1002/lpor.201300043 (2013).

12 Nemilentsau, A. M. Scattering of the near field of an electric dipole by a single-wall carbon nanotube. *Journal of Nanophotonics* **4**, doi:10.1117/1.3416909 (2010).

13 Taylor, R., Hnatovsky, C. & Simova, E. Applications of femtosecond laser induced self-organized planar nanocracks inside fused silica glass. *Laser & Photonics Review* **2**, 26-46, doi:10.1002/lpor.200710031 (2008).

14 Craig Jr, R. R. & Taleff, E. M. *Mechanics of materials*. (John Wiley & Sons, 2020).

15 Fleming, J. W. *et al.* *Handbook of optical materials*. (CRC press, 2018).
